# Noise2Average: An iterative residual learning strategy for image denoising without clean data

**DOI:** 10.1162/IMAG.a.1163

**Published:** 2026-03-24

**Authors:** Zihan Li, Ziyu Li, Berkin Bilgic, Kui Ying, David H. Salat, Jonathan R. Polimeni, Hongen Liao, Susie Y. Huang, Qiyuan Tian

**Affiliations:** School of Biomedical Engineering, Tsinghua University, Beijing, P.R. China; Oxford Centre for Integrative Neuroimaging, FMRIB, Nuffield Department of Clinical Neurosciences, University of Oxford, Oxford, United Kingdom; Athinoula A. Martinos Center for Biomedical Imaging, Department of Radiology, Massachusetts General Hospital, Charlestown, MA, United States; Department of Radiology, Harvard Medical School, Boston, MA, United States; Harvard-MIT Program in Health Sciences and Technology, Massachusetts Institute of Technology, Cambridge, MA, United States; Department of Engineering Physics, Tsinghua University, Beijing, P.R. China

**Keywords:** magnetic resonance imaging, diffusion tensor imaging, self-supervised learning, transfer learning

## Abstract

Magnetic resonance imaging (MRI) is a widely adopted non-invasive imaging tool for both clinical diagnosis and neuroscientific research. Nonetheless, the quality of MRI is often hampered by noise. Supervised deep learning-based denoising has proven to outperform conventional methods but requires high-signal-to-noise ratio (SNR) reference data for supervising the training, which considerably reduces its practical feasibility. To address this challenge, we propose a new iterative residual learning strategy entitled “Noise2Average” for denoising MRI data with multiple repetitions, which can be combined with transfer learning for subject-specific self-supervised training. Noise2Average learns to map each noisy repetition to the average of all noisy repetitions by fine-tuning parameters of a pre-trained convolutional neural network (CNN) and recovers higher SNR by averaging all denoised results at the first iteration, and performs this supervised residual learning-based denoising process repeatedly with the denoising results from the previous iteration as the training target for several iterations. The efficacy of Noise2Average is systematically and comprehensively demonstrated on four types of commonly acquired MRI data, including two or more consecutively acquired highly accelerated T_1_-weighted (T1w) image volumes, two T1w image volumes acquired with different echo times, two diffusion-weighted image (DWI) volumes acquired with opposite phase encoding directions, and two DWI volumes synthesized using different sets of DWI volumes from a diffusion tensor imaging (DTI) scan. Quantitative evaluations show that Noise2Average preserves more image sharpness and textural details and produces more accurate quantitative microstructural metrics from DTI signal modeling than the classic Noise2Noise method and conventional benchmark denoising methods BM4D and AONLM, with denoising performance slightly inferior to that of supervised learning-based denoising method. By reducing the requirement for training data and scan time, Noise2Average substantially increases the feasibility and accessibility of deep learning-based denoising methods for MRI and potentially benefits a wider range of clinical and neuroscientific studies.

## 1 Introduction

Magnetic resonance imaging (MRI) is an important non-ionizing and non-invasive medical imaging tool widely adopted in research and clinical settings. MRI provides detailed images of internal body structures at millimeter spatial resolution and offers a variety of contrast mechanisms to probe different properties of tissues. Anatomical MRI provides superior contrast between healthy and/or pathological tissues at high spatial resolution. For example, T_1_-weighted (T1w) anatomical MRI is routinely used to segment brain structures for morphological analysis (Abdel-Aty et al., 2007; Bederson et al., 1986; Fischl, 2012; Fischl et al., 1999; Lemieux et al., 1998; Pacifico et al., 2011). Diffusion MRI infers from the hindrance and restriction of the diffusive motion of water molecules within the tissue, enabling the measurement of tissue microstructure, such as local axonal orientations for tracking long-range trajectories of white matter fiber pathways (Le Bihan, 2003; Le Bihan et al., 2006). Diffusion tensor MRI (DTI)-based tractography is routinely used for presurgical planning in clinical practice (Avecillas-Chasin et al., 2015; Berman, 2009; Costabile et al., 2019). Other DTI metrics such as fractional anisotropy (FA) and mean diffusivity (MD) are useful for characterizing tissue microstructure and monitoring microstructural alterations associated with development, aging, neurodegeneration, plasticity, and brain disorders (Burzynska et al., 2010; Clark et al., 2011; Qiu et al., 2008).

Nonetheless, the quality of MR images is often hampered by noise, which not only confounds the qualitative interpretation of MR images for clinical diagnosis but also reduces the accuracy and precision of subsequent analytic tasks, such as image segmentation, registration, voxel-wise signal modeling, and analysis of four-dimensional (4D) functional and diffusion MRI data. The signal-to-noise ratio (SNR) level of MR images acquired on low-field MRI scanners, using low-sensitivity receive coils, at high spatial resolution, or with specialized MRI contrast preparation mechanisms, is by nature low. For example, diffusion MRI creates image contrast through signal attenuation that depends on the apparent diffusion coefficient of water molecules, reflecting tissue microstructure and diffusion barriers (Bammer, 2003), while MR spectroscopy acquires signals from inherently low concentration metabolites (Mekle et al., 2009). Due to reduced Fourier averaging of noise, MR images acquired with high acceleration factors also suffer from low SNR, even though the state-of-the-art fast imaging methods can reconstruct images without structural artifacts and g-factor noise amplification (Bilgic et al., 2015). High-performance hardware systems improve MR image quality, which, however, are costly and may not be accessible. Acquiring and averaging multiple repetitions of the same acquisition (or image volumes from different time points for functional MRI and different diffusion-encoding directions for diffusion MRI) is a common practice to improve SNR but increases the scan time and cost and may cause discomfort in subjects (Eshed et al., 2007), especially for children, elderly subjects, and patients who cannot remain still for a long time.

Image denoising provides an alternative approach to improve MR image quality, which aims to restore a clean image with higher SNR from a degraded observation corrupted by noise. In the field of image processing, numerous denoising methods (Aharon et al., 2006; Chang et al., 2000; Dabov et al., 2006; Fischl & Schwartz, 1997; Perona & Malik, 1990) and their extensions for volumetric data (Bazin et al., 2019; Coupé Hellier, et al., 2008; Coupé, Yger, et al., 2008; Gerig et al., 1992; Maggioni et al., 2012; Manjón et al., 2010) have been proposed, which can be directly applied to denoising MRI data. Many denoising techniques tailored for specific MRI modalities have also been developed. For example, diffusion-weighted images (DWIs) along multiple diffusion-encoding directions in DTI are highly correlated and the inter-image redundancy can be exploited for denoising DWIs (Fadnavis et al., 2020; Veraart, Fieremans, et al., 2016; Veraart, Novikov, et al., 2016). The denoising is also achieved by regularizing the MR image reconstruction from k-space data (Haldar et al., 2013; Hu et al., 2020; Varadarajan & Haldar, 2015). More recently, deep learning using convolutional neural networks (CNNs) has been demonstrated to be a superior technology for image denoising (Zhang et al., 2017) and is widely adopted in biomedical imaging (Li et al., 2022; Tian, Zaretskaya, et al., 2021; Zhu et al., 2025).

Nonetheless, most deep learning-based denoising methods require high-SNR reference data for supervising the training of the adopted CNN, which significantly reduces their practical feasibility and accessibility. High-SNR reference data are often obtained by averaging numerous repetitions of images, which might be difficult to acquire in practice or can only be acquired on a few subjects due to the prolonged scan time with the more likely appearance of subject motion and associated image artifacts. It is also hard to obtain high-SNR reference images by data averaging for some applications such as functional MRI since brain dynamics are constantly changing.

A novel learning strategy entitled Noise2Noise (Lehtinen et al., 2018) addresses this problem, which trains a CNN to learn to map one noisy image to another repetition of the noisy image instead of the high-SNR image. It is proved that the learned CNN parameters remain unchanged if certain statistics of noisy target values match those of high-SNR target values (e.g., the expectation for L2 minimization). Intuitively, the CNN in Noise2Noise acts as an image approximator but cannot effectively approximate the random noise in the image and, therefore, the CNN output becomes approximately free of noise. While the application of Noise2Noise in natural image processing is often limited by the difficulty of acquiring paired noisy images, leading to the development of alternative approaches that can operate with single noisy images (Ma et al., 2025; Moran et al., 2020; Quan et al., 2020), Noise2Noise still has a wide range of applications in MRI where repeated data are readily available, such as the multiple repetitions of images for averaging and multiple images from different elements of a phased array coil. In diffusion MRI, pairs of DWIs with opposite phase-encoding directions are often acquired to compensate for susceptibility-induced geometric distortion and signal dropout and pile-up. Furthermore, since numerous DWIs are acquired along different diffusion-encoding directions, repetitions of images could be synthesized through diffusion signal modeling (Tian, Li, et al., 2022).

Unfortunately, one essential assumption of Noise2Noise is that the two repetitions of the data only differ in noise. This assumption cannot be satisfied in practice due to the presence of subject motion and associated spatially and temporally varying image artifacts that cannot be perfectly accounted for by artifact correction and image co-registration. The mismatch in geometry and signal intensity leads to image blurring, a well-known problem associated with using voxel-wise errors as the loss for training CNNs (Ledig et al., 2017).

In this study, we show the degraded performance of Noise2Noise on empirical MRI data and propose a new learning strategy entitled “Noise2Average” to address this challenge. Rather than mapping one noisy image to another repetition, the CNN of Noise2Average maps a noisy image to the residual between it and the average of the two (or more) noisy images at the first iteration and to the residual between it and the denoised image from the previous iteration at subsequent iterations. We demonstrate the efficacy of Noise2Average for the case of two repetitions of empirical MRI data that are commonly acquired, including (1) consecutively acquired highly accelerated T1w image volumes, (2) T1w image volumes acquired with different echo times in multi-echo sequences, (3) DWI volumes acquired with opposite phase encoding directions, and (4) synthesized DWI volumes from subsets of a DTI scan. We systematically and quantitatively compare the denoising performance of the proposed Noise2Average, Noise2Noise, supervised learning-based denoising with external high-SNR reference data, and two conventional benchmark denoising methods block matching and 4D filtering (BM4D) (Dabov et al., 2007; Maggioni et al., 2012) and adaptive optimized nonlocal means (AONLM) (Manjón et al., 2010), as well as those designed for diffusion MRI, including the Marchenko–Pastur principal component analysis (MPPCA) (Veraart, Fieremans, et al., 2016), local complex principal component analysis (LCPCA) (Bazin et al., 2019), and Patch2Self (Fadnavis et al., 2020), in terms of the image similarity and the accuracy of resultant DTI metrics for DWI denoising compared with the ground truth. We show that Noise2Average is similar to supervised denoising and outperforms other denoising methods. We also demonstrate the value of Noise2Average in denoising sub-millimeter isotropic resolution brain imaging data, including T1w data at 250 µm and 600 µm isotropic resolution and DTI data at 760 µm isotropic resolution. Because of its superior denoising performance, subject-specific training without the need for external high-SNR reference data, and rapid training and inference, we expect that Noise2Average can be more easily deployed for faster MRI with higher resolution and SNR to benefit a wider range of neuroscientific and clinical applications.

## 2 Noise2Average Methodology

**Fig. 1.**
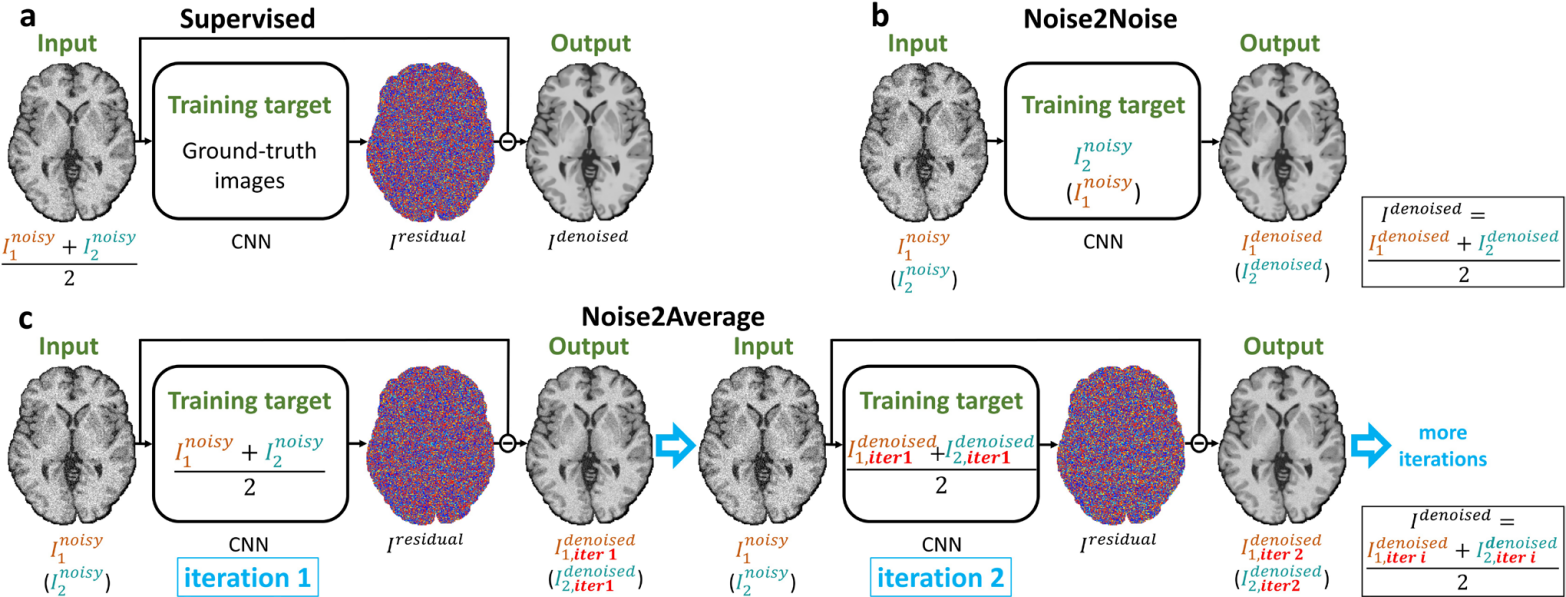
Learning strategies. Learning strategies of supervised (a), Noise2Noise (b), and Noise2Average (c) denoising are illustrated for the case of two repetitions of noisy images. Supervised denoising trains a CNN to map the average of the two noisy images to its residual image compared with the ground-truth image with high signal-to-noise ratio (SNR) (a). Noise2Noise trains a CNN to map one noisy image to the other noisy image (b). Noise2Average performs supervised residual learning iteratively, with the training target as the average of the two noisy images with slightly higher SNR for iteration 1, and the average of denoised images from iteration *i*-1 for iteration *i* (*i* = 2, 3, 4,…) (c).

Noise2Average employs an iterative supervised residual learning strategy without the need for high-SNR images as the training target (Supplementary Derivations). Intuitively, when *n* repetitions of noisy images are available, Noise2Average trains a CNN to map each noisy image to its residual image (containing noise and differences from all sources) compared with the average of all *n* noisy images with higher SNR (*n* = 2 illustrated in Fig. 1c), following the standard supervised learning-based denoising method (Fig. 1a). Each denoised image exhibits image quality (e.g., image SNR and sharpness) similar to that of the *n*-repetition averaged image due to the superior denoising performance of the CNN but with different noise observations. Therefore, the average of *n* denoised images recovers a higher SNR that is comparable with the average of *n*×*n* repetitions of raw noisy images. This residual learning process is repeatedly performed for the subsequent iterations, with the resultant denoised image from the previous iteration as the training target. In the ideal case, if the CNN can match the image quality of the input noisy image to the target image perfectly, the denoised image from iteration *i* (*i* = 1, 2, 3,…) is equivalent to the average of *n^i+1^* raw noisy images. However, in practice, the CNN introduces blurring to denoised images and, therefore, the quality of the target image for training at subsequent iterations continues to decrease. Consequently, the image quality, such as the image sharpness of the resultant denoised image, may decrease after a certain number of iterations.

Any CNN architecture for denoising can be used in Noise2Average, such as the commonly adopted plain network (Kim et al., 2016; Simonyan & Zisserman, 2014) and U-Net (Falk et al., 2019). In most cases, the data from a single subject are not adequate for optimizing the parameters of the adopted CNN for subject-specific training, unless the CNN only has a small number of parameters (e.g., with a very shallow architecture) or the spatial resolution of the image volume is high, containing hundreds of millions of voxels that can be used for the optimization. Because residual learning is used and the CNN is more generalizable, transfer learning can address this challenge. Specifically, the CNN can be pre-trained on a large amount of data and then fine-tuned on the data of a particular subject for denoising, which substantially reduces the training time. The image contrast and SNR level of pre-training data can be slightly different from those of the data for denoising (i.e., acquired with distinct hardware systems and imaging sequences and protocols). Therefore, the pre-training data may be easily obtained from large public databases or simulation. When sufficient data from multiple subjects are available in a study, the Noise2Average CNN can be trained from random initialization without requiring additional pre-training data. Furthermore, this multi-subject trained model can be fine-tuned on individual subject data to achieve subject-specific optimization and potentially enhanced denoising performance.

## 3 Experiments

### 3.1 Evaluation data

#### 3.1.1 MGH T_1_-weighted wave-MPRAGE data

Highly accelerated brain imaging data (0.8 mm isotropic resolution, 3 × 3 acceleration factor, 10 repetitions per subject) were acquired using a 3D T1w Wave-CAIPI magnetization-prepared rapid gradient-echo (MPRAGE) sequence (Longo et al., 2020; Polak et al., 2018, 2019) on 10 healthy subjects on a whole-body 3-Tesla MAGNETOM Skyra scanner (5 subjects) and a whole-body 3-Tesla MAGNETOM Prisma scanner (5 subjects) (Siemens Healthineers, Erlangen, Germany) using a vendor-supplied 32-channel receive coil at the Massachusetts General Hospital (MGH) Martinos Center for Biomedical Imaging with Institutional Review Board approval and written informed consent of the volunteers. The sequence parameters were repetition time = 2,530 ms, echo time = 3.65 ms, inversion time = 1,100 ms, excitation flip angle = 7° (non-selective), bandwidth = 200 Hz/pixel, field of view = 256 × 256 × 192 mm^3^, slice thickness = 0.8 mm, matrix size = 320 × 320, 240 sagittal slices, phase encoding turbo factor = 3, 16 wave cycles with a maximum gradient amplitude 9 mT/m and a maximum slew rate 160 mT/m/ms, acquisition time = 97 s per repetition.

For each subject, the 10 repetitions of data were co-registered using the “mri_robust_template” function (Reuter et al., 2010) from the FreeSurfer software package (Dale et al., 1999; Fischl, 2012; Fischl et al., 1999) (version 6.0, https://surfer.nmr.mgh.harvard.edu). The first two repetitions were used for denoising. The average of all 10 repetitions was used as the ground-truth image. Data of all 10 subjects were used for evaluation.

#### 3.1.2 MGH T_1_-weighted ME-MPRAGE data

Ultra-high-resolution brain imaging data (0.6 mm isotropic, 6 repetitions per subject) were acquired using a 3D multi-echo MPRAGE (ME-MPRAGE) sequence (van der Kouwe et al., 2008) on nine healthy subjects on a whole-body 3-Tesla scanner (MAGNETOM Trio Tim system, Siemens Healthcare, Erlangen, Germany) using a vendor-supplied 32-channel receive coil at the MGH Martinos Center with institutional review board approval and written informed consent of the volunteers. In order to minimize the number of partition encoding steps, a slab-selective oblique-axial acquisition using a 13 ms FOCI adiabatic inversion pulse (Hurley et al., 2010) and acceleration factor of 2 in the partition direction was employed (Polak et al., 2018; Tisdall et al., 2013; van der Kouwe et al., 2014). The sequence parameters were repetition time = 2,510 ms, echo times = 2.88/5.6 ms, inversion time = 1,200 ms, excitation flip angle = 7°, bandwidth = 420 Hz/pixel, echo spacing = 8.4 ms, 224 axial slices, matrix size = 400 × 304, slice thickness = 0.6 mm, field of view = 240 mm × 182 mm, generalized autocalibrating partial parallel acquisition (GRAPPA) factor = 2, acquisition time = 10.7 min per repetition. The two image volumes of each repetition with different echo times were combined using root mean square combination.

**Fig. 2.**
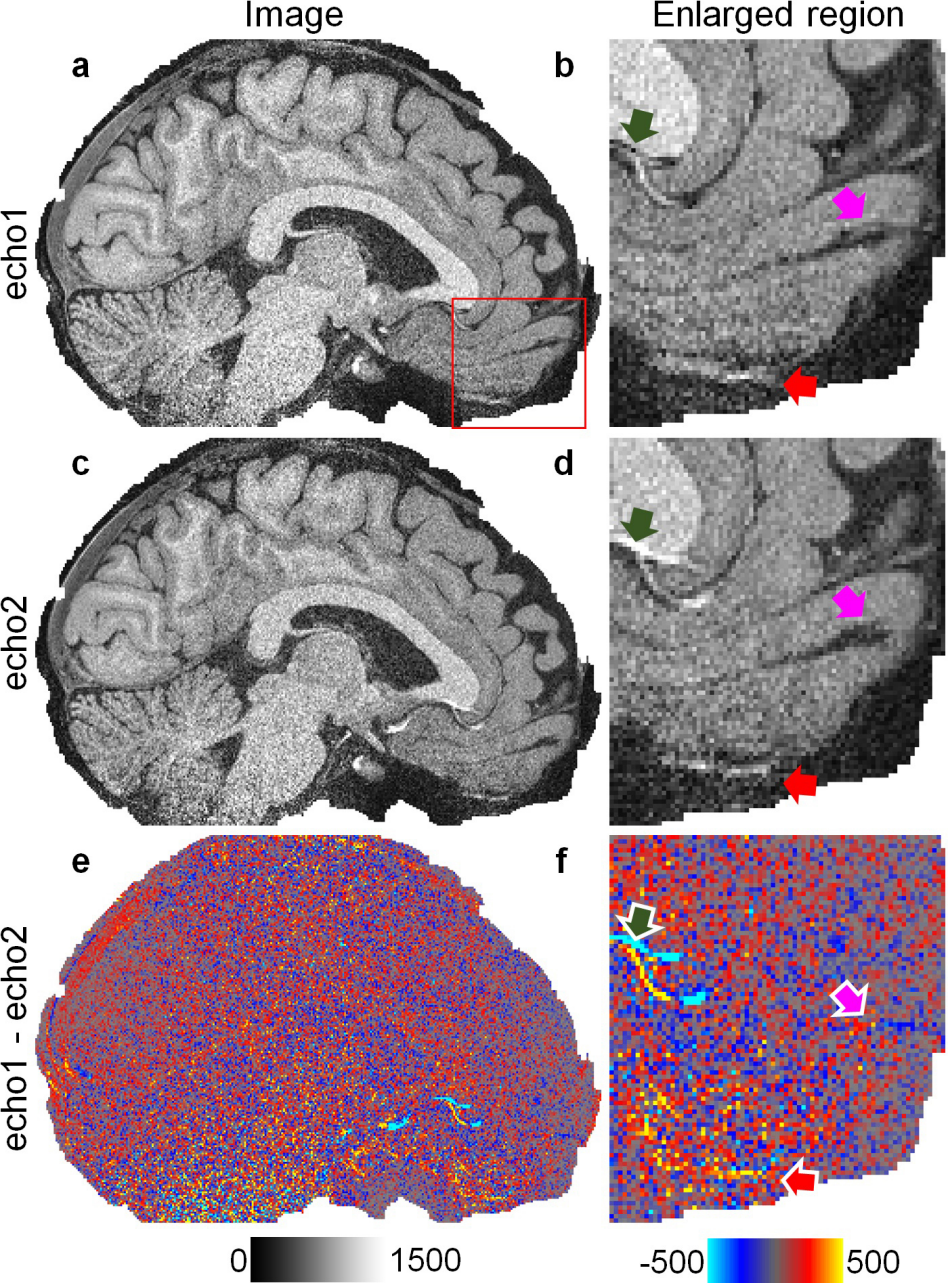
Multi-echo T1w images. Exemplary sagittal images with two different echo times (a, c) and the residual map between them is displayed (e), along with enlarged views (b, d, f) of a region of interest (the red box in a). Arrowheads highlight regions where the two images with different echo times differentiate not only in the noise observation.

Because the profile of the radiofrequency pulse for the slab excitation fell off at the slab boundaries, image intensities were lower toward the superior and inferior parts of the brain. For each combined volume, the intensity bias was, therefore, corrected using the unified segmentation routine (Ashburner & Friston, 2005) implementation in the Statistical Parametric Mapping software package (SPM, https://www.fil.ion.ucl.ac.uk/spm) with a full-width at half-maximum (FWHM) of 30 mm and a sampling distance of 2 mm (Uwano et al., 2014). The derived bias maps were also applied to correct each single echo image volume (Fig. 2a, c). The residual maps between the two image volumes with different echo times (Fig. 2e, f) exhibited not only random noise but also biases reflecting the underlying anatomy such as those near the air–tissue interface in the frontal lobe (Fig. 2 arrowheads) and the dura.

For each subject, the combined image volumes from the second to sixth repetition were registered to the combined volume from the first repetition using FreeSurfer’s “mri_robust_template” function (Reuter et al., 2010). The first repetition consisting of image volumes with two echo times was used for denoising. The average of combined volumes from all six repetitions was used as the ground-truth image. Data from five randomly selected subjects were used for evaluation, while the data from the other four subjects were used for the training of supervised learning-based denoising.

In addition, Noise2Average was also performed to denoise the data of an evaluation subject by mapping each echo-combined image volume to the average of echo-combined volumes from all six repetitions.

#### 3.1.3 HCP in aging (HCP-A) diffusion MRI data

Diffusion MRI data of 30 healthy subjects between 35 and 90 years of age were acquired at the MGH Martinos Center with approval from the institutional review board and written informed consent from all participants as part of the Lifespan Human Connectome Project in Aging (HCP-A) (Bookheimer et al., 2019; Harms et al., 2018). The acquisition details were described in previous studies (Bookheimer et al., 2019; Harms et al., 2018). Briefly, the diffusion data were acquired at 1.5 mm isotropic resolution, with 93 DWI volumes along uniform diffusion-encoding directions at b = 1,500 s/mm^2^ and 14 interleaved b = 0 image volumes. Each volume was acquired with anterior-posterior and posterior-anterior phase-encoding directions. It is worth mentioning that the 2D echo planar imaging (EPI) acquisition employed a very high simultaneous multi-slice factor of four and, therefore, did not use in-plane acceleration, resulting in strong susceptibility-induced geometric distortion and signal pile-up and dropout near the air-tissue interface.

**Fig. 3.**
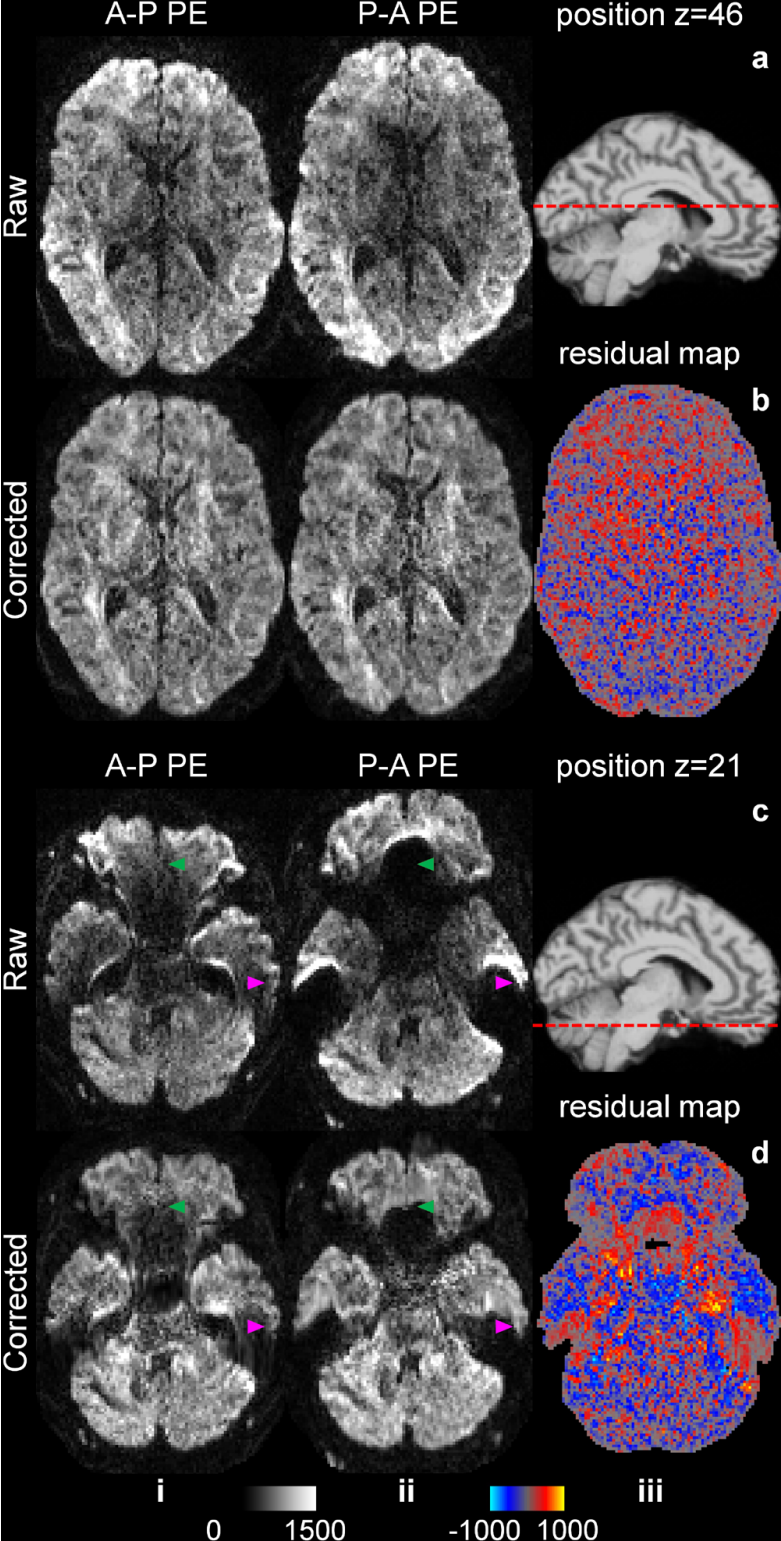
Diffusion-weighted images with opposite phase-encoding directions. Exemplary axial image slices acquired with opposite phase-encoding (PE) directions (i.e., anterior-posterior (AP) and posterior-anterior (PA)) (i, ii) before (a, c) and after (b, d) geometric distortion and signal intensity correction using the “topup” function of FSL through the middle brain (a, b) and brain regions near air-tissue boundaries (c, d, green and magenta arrowheads) of a representative subject from the HCP-A data are shown. The positions of the two image slices are displayed on top of a sagittal T1w image slice, indicated by red dotted lines (a, iii and c, iii). Residual maps between corrected images with opposite PE directions are shown (b, iii and d, iii).

Diffusion data were corrected for eddy current and susceptibility-induced distortions and co-registered using the “topup” and “eddy” functions from the FMRIB Software Library (FSL) software package (Andersson et al., 2003; Andersson & Sotiropoulos, 2016; Jenkinson et al., 2012; Smith et al., 2004) (https://fsl.fmrib.ox.ac.uk). The corrected image volumes acquired with anterior–posterior and posterior–anterior phase-encoding directions were averaged. The residual maps between the anterior–posterior and posterior–anterior image pairs exhibited random noise in most brain regions (Fig. 3b, iii) but contained structural biases in regions near the air–tissue interface with severe susceptibility-induced image artifacts (Fig. 3d, iii) even after FSL’s “*topup*” correction, which made the assumption of Noise2Noise invalid.

Diffusion tensor model fitting was performed on the 107 averaged image volumes of each subject using FSL’s “dtifit” function with ordinary least squares regression to derive the ground-truth diffusion tensors and DTI metrics such as the primary eigenvectors (V1), fractional anisotropy (FA), and mean diffusivity (MD). Volumetric segmentation was performed on the T1w MRI data (0.8 mm isotropic resolution) using FreeSurfer’s “recon-all” function. For each subject, the T1w and the mean diffusion b = 0 image volumes were affinely co-registered (degree of freedom equal to 12) using boundary-based registration implemented in FreeSurfer’s “bbregister” function. FreeSurfer’s volumetric segmentation results (i.e., “aparc+aseg”) were then resampled to the diffusion image space using nearest neighbor sampling with the derived affine transformation to generate brain tissue masks (i.e., gray matter and white matter, excluding cerebrospinal fluid) for evaluating DTI metrics from different methods.

A subset of 14 corrected image volumes consisting of the first b = 0 image volume and the first 6 DWI volumes (the acquisition was optimized such that any first *N* directions were uniformly distributed) acquired with anterior-posterior and posterior-anterior phase-encoding directions were selected for denoising. The ground-truth b = 0 image volume was obtained by averaging all 28 b = 0 image volumes. The ground-truth DWI volumes were derived using the tensor model from the ground-truth diffusion tensors fitted using all data of each subject. Data from 10 randomly selected subjects were used for evaluation, while the data from the other 20 subjects were used for the training of supervised learning-based denoising.

#### 3.1.4 WU-Minn-Ox HCP diffusion MRI data

Pre-processed diffusion MRI data (1.25 mm isotropic resolution, 18 b = 0 image volumes, 90 DWI volumes along uniform diffusion-encoding directions at b = 1,000 s/mm^2^) of 30 unrelated healthy subjects from the HCP, WU-Minn-Ox Consortium (https://www.humanconnectome.org) were used. The acquisition details were described in previous studies (Glasser et al., 2013; Sotiropoulos et al., 2013; Ugurbil et al., 2013). Diffusion tensor model fitting was performed on the 108 image volumes of each subject using FSL’s “dtifit” function to derive the ground-truth diffusion tensors and DTI metrics such as V1, FA, and MD. Moreover, volumetric segmentation results (i.e., “aparc+aseg”) from the FreeSurfer reconstruction using T1w data of each subject were resampled to the diffusion image space using nearest neighbor sampling with an identity transformation matrix (since T1w and diffusion data were already co-registered) to generate brain tissue masks for evaluating DTI metrics from different methods.

For each subject, 2 b = 0 image volumes and 2 sets of 6 DWI volumes along optimized diffusion-encoding directions that minimize the condition number of the diffusion tensor transformation matrix while being as uniform as possible were used for denoising (a total of 14 image volumes). For the selection of the DWI volumes, the six optimal directions from the DSM scheme (Skare et al., 2000) (associated with a condition number of 1.3228) were randomly rotated and the six nearest directions were chosen if their associated condition numbers were lower than 1.6. Then 2 out of 46 chosen sets were randomly picked many times, and the 12 selected directions with the lowest electrostatic potential energy (Jones, 2004) were chosen to ensure that they were as uniformly distributed on a sphere as possible.

For each set of 6 DWI volumes, diffusion tensor model fitting was performed using FSL’s “dtifit” function along with the average b = 0 image volume to derive the diffusion tensors, which were then used to synthesize DWI volumes along the 12 selected diffusion-encoding directions. The noise and artifacts were not amplified during the diffusion tensor model fitting which was well conditioned due to the carefully designed diffusion encoding directions. In this way, 2 repetitions of 1 b = 0 image volume and 12 DWI volumes with identical contrast and different noise observations were generated for Noise2Noise and Noise2Average denoising.

The ground-truth b = 0 image volume was obtained by averaging 18 b = 0 image volumes. The ground-truth DWI volumes were synthesized using the tensor model from the ground-truth diffusion tensors fitted using all data of each subject. Data from 10 randomly selected subjects were used for evaluation, while the data from the other 20 subjects were used for the training of supervised denoising.

#### 3.1.5 OVGU 7T T_1_-weighted data

The public ultra-high-resolution brain imaging data of a healthy subject (0.25 mm isotropic, eight repetitions, 53 min per repetition) acquired using a 3D MPRAGE sequence (Mugler III & Brookeman, 1990) on a whole-body 7-Tesla MRI scanner with prospective motion correction were used. The acquisition details were described in the previous studies (Lüsebrink et al., 2017). The image intensity bias of each repetition was removed. A combined image volume obtained by non-linearly co-registering and averaging all eight repetitions was provided. Each individual repetition was available, but each co-registered repetition was not provided.

Each repetition was non-linearly registered to the provided combined image volume using the “reg_f3d” function (default parameters, spline interpolation) from the NiftyReg software (https://github.com/KCL-BMEIS/niftyreg) (Modat et al., 2010, 2014). Co-registered image volumes were used for Noise2Average denoising.

#### 3.1.6 MGH gSlider-SMS diffusion MRI data

The public ultra-high-resolution diffusion MRI data of a healthy subject (0.76 mm isotropic) acquired using the gSlider-SMS sequence (Liao et al., 2019, 2020; Setsompop et al., 2018) on the whole-body MGH-USC 3-Tesla Connectom scanner equipped with a 64-channel phased-array coil (Keil et al., 2013) were used. The acquisition details were described in the previous studies (Wang et al., 2021). The provided data were already corrected for eddy current and susceptibility-induced distortions and co-registered using the “topup” and “eddy” functions of FSL. A subset of the data consisting of the first 3 b = 0 image volumes and first 30 DWI volumes at b = 1,000 s/mm^2^ (the acquisition was optimized such that any *N* first directions were uniformly distributed) acquired with anterior–posterior and posterior–anterior phase-encoding directions (18.3 min acquisition time) was used for Noise2Average denoising. Diffusion tensor model fitting was performed on the raw and denoised data using FSL’s “dtifit” function to derive DTI metrics.

#### 3.1.7 Simulation data

High-quality T1w MRI data of 10 subjects (1 mm isotropic) from the publicly available comprehensive diffusion MRI dataset (CDMD) (Tian, Fan, et al., 2022) acquired using a 3-dimensional ME-MPRAGE sequence with echo times (TE) of 1.15, 3.03, 4.89, and 6.75 ms, ensuring high SNR and serving as reliable ground-truth data were used. Specifically, the T1w data of each subject were affinely transformed 5 times, by random translations (up to 10 voxels in each direction), random rotations (within ±3° around each axis), and random scaling in the left–right direction (scaling factor: 1.0 to 1.4), to generate 5 repetitions of noisy brain image volumes. Subsequently, zero-mean Gaussian noise (noise level: σ × standard deviation of brain voxel intensities) was added to the transformed image volumes. Finally, the noise-corrupted images were registered to the original ground-truth T1w data using FSL’s “flirt” function, generating five repetitions of co-registered noisy image volumes for denoising. The experiments evaluated both mild (σ = 0.3) and heavy (σ = 0.5) noise levels.

### 3.2 Pre-training data

#### 3.2.1 WU-Minn-Ox HCP T_1_-weighted data

Pre-processed T1w MRI data (0.7 mm isotropic resolution) of 20 young adults from WU-Minn-Ox HCP were used for pre-training CNNs. For each high-SNR image volume, two simulated noisy image volumes were generated by adding Gaussian noise (μ = 0, σ = 0.5 × standard deviation of brain voxel intensities). The simulated noisy data and the ground-truth data were used for pre-training CNNs of Noise2Noise and Noise2Average.

#### 3.2.2 MGH-USC HCP diffusion MRI data

Pre-processed diffusion MRI data (1.5 mm isotropic resolution, 18 b = 0 image volumes, 64 DWI volumes along uniform diffusion-encoding directions at b = 1,500 s/mm^2^) of 35 subjects from MGH-USC HCP were used in this study. Diffusion tensor model fitting was performed on the data of each subject using FSL’s “dtifit” function to derive the ground-truth diffusion tensors. For each subject, the ground-truth b = 0 image volume was generated by averaging all 18 b = 0 image volumes. The ground-truth DWI volumes along the 6 selected diffusion-encoding directions for the HCP-A diffusion data, the 12 selected diffusion-encoding directions for the WU-Minn-Ox HCP diffusion data, and 30 selected diffusion-encoding directions for MGH gSlider-SMS diffusion data were synthesized using the tensor model from ground-truth diffusion tensors.

For each set of 1 ground-truth b = 0 image and 6, 12, or 30 DWI volumes, 2 sets of simulated noisy image volumes were generated by adding Gaussian noise to each volume (μ = 0, σ = 0.1 × standard deviation of brain voxel intensities for b = 0 image volume, μ = 0, σ = 0.3 × standard deviation of brain voxel intensities for each DWI volume). The simulated noisy data and the ground-truth data were used for pre-training CNNs of Noise2Noise and Noise2Average.

### 3.3 Neural network implementation

**Fig. 4.**
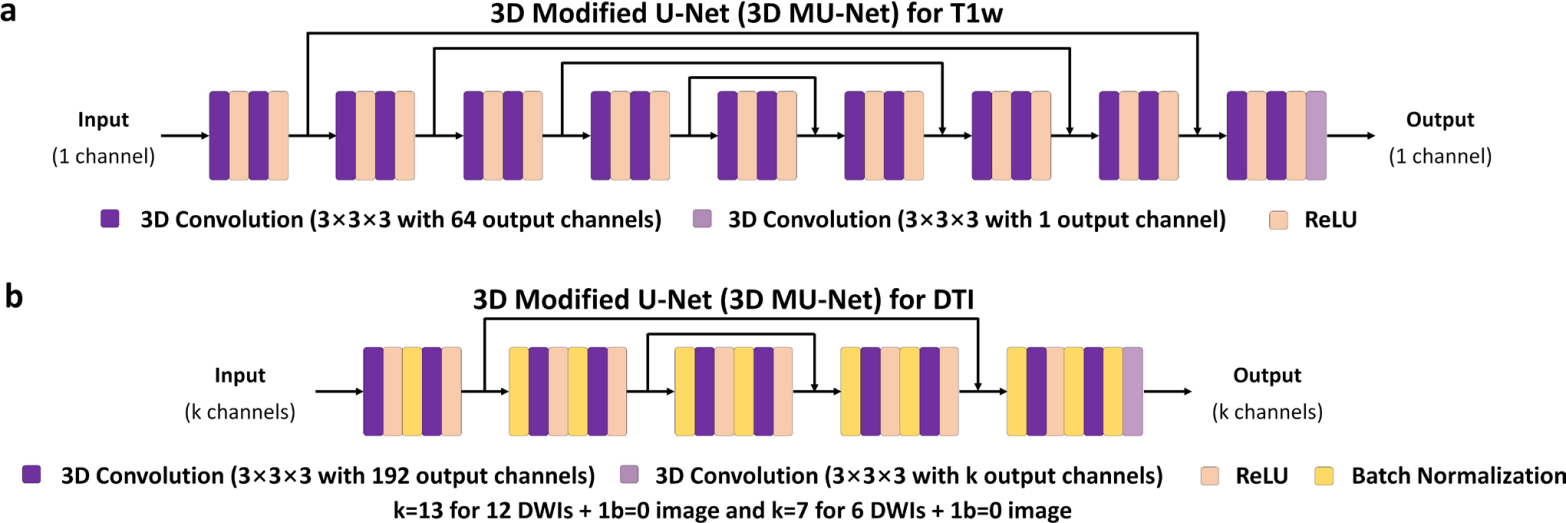
Convolutional neural networks. 3D modified U-Nets (MU-Nets) excluding pooling and up-sampling layers are adopted in this study. For denoising T1w images at higher spatial resolution, a deeper MU-Net (18 layers) with a larger receptive field is employed (a), with 64 channels at intermediate layers. For denoising diffusion images at lower resolution but with much more image channels, a shallower MU-Net (10 layers) is employed, with 192 channels at intermediate layers and batch normalization before every 3D convolution layer (b).

Modified U-Nets (MU-Nets) incorporating 3D convolution were employed for CNN-based denoising in this study, with the pooling and up-sampling layers excluded (Li et al., 2022). The 3D convolution kernels (3 × 3 × 3 size, 1 × 1 × 1 stride) provided larger receptive fields than 2D convolution kernels for leveraging more redundant information from an additional spatial dimension as well as avoiding boundary artifacts in the cross-slice direction. For denoising T1w images at higher spatial resolution, a deeper MU-Net (18 layers) with a larger receptive field was employed (Fig. 4a). For denoising diffusion images at lower resolution but with much more image channels, a slightly shallower MU-Net (10 layers) was employed, with 192 channels at intermediate layers and batch normalization before every 3D convolution layer (Fig. 4b).

Input noisy images and associated training targets were standardized by subtracting the mean value and then dividing by the standard deviation of image intensities of voxels within the brain mask from the input images. For diffusion data, the standardization was performed for each image channel independently. The standardization removed inter-subject and inter-channel variations in image intensity. The denoised images from CNNs were transformed back to the original intensity range accordingly.

MU-Nets were implemented using the Keras application programming interface (https://keras.io) with a Tensorflow backend (https://www.tensorflow.org, version 2.2.0). Network training employed the mean squared error (MSE) for T1w data, taking advantage of its theoretical optimality under the zero-mean Gaussian noise assumptions typical of T1w acquisitions. For diffusion data, the mean absolute error (MAE) was used, owing to its robustness against outliers commonly encountered in lower-SNR DWI acquisitions (Tian, Li, et al., 2022). The Adam optimizer (Kingma & Ba, 2014) was used with changes to learning rate outlined in subsequent sections and other parameter values as default. The loss was only calculated within the brain mask to exclude regions that were irrelevant to the brain tissue. Due to the limited GPU memory, image blocks consisting of 80 × 80 × 80 × 1 voxels and 64 × 64 × 64 × *n* voxels (*n* = 7 for HCP-A diffusion data, *n* = 13 for WU-Minn-Ox HCP diffusion data and *n* = 34 for MGH gSlider-SMS diffusion data) were extracted from the T1w data and diffusion data of each subject, respectively, for training (the smallest number of blocks with overlap that could cover the whole brain). The batch size was set to 1. Trained CNNs were applied to denoise image blocks for each subject and the denoised image blocks were then assembled into a whole brain volume with overlapped regions averaged. For Noise2Noise and Noise2Average pre-training and supervised learning, data from 80% randomly selected subjects were used for training while data from the remaining 20% of subjects were used for validation. For Noise2Noise and Noise2Average fine-tuning on data of each subject, 80% randomly selected extracted image blocks were used for training while the remaining 20% blocks were used for validation at each epoch (i.e., Monte Carlo cross-validation).

### 3.4 Data denoising

#### 3.4.1 Noise2Average denoising

MU-Nets of Noise2Average were pre-trained and validated on WU-Minn-Ox HCP T1w data and MGH-USC HCP diffusion data with simulated noisy images as input and ground-truth images as target for 20 epochs with a learning rate of 0.0001 and another 20 epochs with a reduced learning rate of 0.00001. The pre-training took 9.5 h for T1w data and 12.8 and 16.4 h for 6- and 12-direction diffusion data, respectively. Then they were fine-tuned on the data of each subject for evaluation using the MGH T1w Wave-MPRAGE data, MGH T1w ME-MPRAGE data, HCP-A diffusion data, and WU-Minn-Ox HCP diffusion data. The fine-tuning was performed using a learning rate of 0.00001. The numbers of fine-tuning epochs for each iteration were 10, 10, 20, and 20, respectively. It is noteworthy that due to the contrast differences between images with different echo times from MGH T1w ME-MPRAGE data (Fig. 2f), two MU-Nets were fine-tuned separately using image volumes from each echo time as the input.

#### 3.4.2 Noise2Noise denoising

The pre-training and fine-tuning procedures for Noise2Noise followed the same protocol as Noise2Average, but used another repetition of the noisy image(s) as the training target. Denoised results from each repetition were averaged at the end. Similarly, two MU-Nets were fine-tuned separately using image volumes from each echo time as the input for MGH T1w ME-MPRAGE data.

#### 3.4.3 Supervised denoising

MU-Nets for supervised learning-based denoising were trained and validated on the data of 4, 20, and 20 subjects for MGH T1w ME-MPRAGE data, HCP-A diffusion data, and WU-Minn-Ox HCP diffusion data, in a similar way to the Noise2Average pre-training. The two noisy repetitions were first averaged to serve as the input to the CNN.

#### 3.4.4 BM4D denoising

BM4D was an extension of the state-of-the-art BM3D denoising method for volumetric data, which was employed to denoise the T1w and diffusion data using the publicly available MATLAB-based software package (https://webpages.tuni.fi/foi/GCF-BM3D). The two noisy repetitions were first averaged to serve as the input to BM4D for MGH T1w Wave-MPRAGE data, MGH T1w ME-MPRAGE data, and HCP-A diffusion data. For WU-Minn-Ox HCP diffusion data, BM4D was applied to denoise raw acquired image volumes. BM4D was set to estimate the standard deviation of Rician noise and perform collaborative Wiener filtering and was performed using the “modified profile” option and default values for other parameters.

#### 3.4.5 AONLM denoising

AONLM improved upon the classic NLM method by adapting to spatially varying noise levels (Coupé, Yger, et al., 2008; Manjón et al., 2010), which was employed to denoise the T1w and diffusion data. The two repetitions were first averaged to serve as the input to AONLM for MGH T1w Wave-MPRAGE data, MGH T1w ME-MPRAGE data, and HCP-A diffusion data. For WU-Minn-Ox HCP diffusion data, AONLM was applied to denoise raw acquired image volumes. AONLM was performed assuming Rician noise with patch radius and search block radius equal to 1 and 3, respectively.

#### 3.4.6 MPPCA denoising

The MPPCA algorithm employs random matrix theory to identify and suppress noise components by analyzing the eigenvalue spectrum of local signal patches (Veraart, Fieremans, et al., 2016). MPPCA was applied to denoise the raw diffusion data from the WU-Minn-Ox HCP using a 5 × 5 × 5 voxel neighborhood and the “full” sampling mode using the NYU MATLAB software package (https://github.com/NYU-DiffusionMRI/mppca_denoise).

#### 3.4.7 LCPCA denoising

The LCPCA algorithm extends overcomplete local PCA to complex-valued, multi-parametric MRI data (Bazin et al., 2019). LCPCA was applied to denoise the raw diffusion data from the WU-Minn-Ox HCP using four local PCA neighborhoods and a local noise level of 2.0 using the NIGHRES software package (https://nighres.readthedocs.io/en/latest/intensity/lcpca_denoising.html) (Huntenburg et al., 2018).

#### 3.4.8 Patch2Self denoising

The Patch2Self algorithm leverages the statistical independence of noise across different DWI volumes, using *n* − 1 volumes to train a regressor that predicts and denoises the target volume without relying on explicit noise models or signal assumptions (Fadnavis et al., 2020). Patch2Self was applied to denoise the raw diffusion data from the WU-Minn-Ox HCP using the default parameters in the DIPY software package (https://docs.dipy.org/dev/examples_built/preprocessing/denoise_patch2self.html) (Garyfallidis et al., 2014).

#### 3.4.9 Noise2Average denoising for ultrahigh resolution data

Noise2Average was also applied to denoise images acquired at ultrahigh sub-millimeter resolution. Specifically, pre-trained MU-Nets were fine-tuned and applied to denoise the 6-repetition MGH T1w ME-MPRAGE data, 8-repetition OVGU 7T T1w data, and 33-diffusion-encoding-direction MGH gSlider-SMS diffusion data of a single evaluation subject from each dataset.

The fine-tuning for MGH T1w ME-MPRAGE data was similar to the process for other datasets (Section 3.4.1), which was performed for 40 epochs at each iteration. The fine-tuning for OVGU 7T T1w data and MGH gSlider-SMS diffusion data was slightly different. For OVGU 7T T1w data, because the image content and texture of each image block (128 × 128 × 128 size) extracted from the extra-large whole brain volume at 0.25 mm isotropic resolution were very different, the fine-tuning was performed for each individual block (i.e., block-specific training), resulting in 125 different MU-Nets for improved performance. The number of fine-tuning epochs for each iteration was 20. For MGH gSlider-SMS diffusion data, because it was challenging to predict the residual maps for all 33 diffusion encoding directions simultaneously, the fine-tuning was performed to predict the residual for each b = 0 image or DWI volume separately, resulting in 33 MU-Nets. Since DWIs were correlated, DWIs from all 33 channels were appended to the input to provide auxiliary information for improving CNN performance (i.e., the size of CNN input was 64 × 64 × 64 × 34). The number of fine-tuning epochs for each iteration was 20.

### 3.5 Result evaluation

#### 3.5.1 Image quality

The MAE, peak SNR (PSNR), and whole brain averaged structural similarity index (SSIM) within the brain mask were computed to quantify the image similarity of raw and denoised images compared with the ground truth. For the calculation, image intensities were standardized and then transformed by adding three and dividing by six, resulting in image intensities ranging approximately between 0 and 1. PSNR and SSIM were calculated using the “psnr” and “ssim” functions of the MATLAB software package, respectively. The group means and standard deviations of the MAE, PSNR, and SSIM across all evaluation subjects for each dataset were calculated.

#### 3.5.2 DTI metrics

For diffusion data, the MAE of five DTI metrics including V1, FA, MD, AD, and RD, within the brain tissue mask excluding cerebrospinal fluid compared with the ground truth was used to quantify the quality of raw and denoised images for diffusion tensor modeling. The group means and standard deviations of MAE within the tissue mask for different DTI metrics across all evaluation subjects for each dataset were calculated.

## 4 Results

**Fig. 5.**
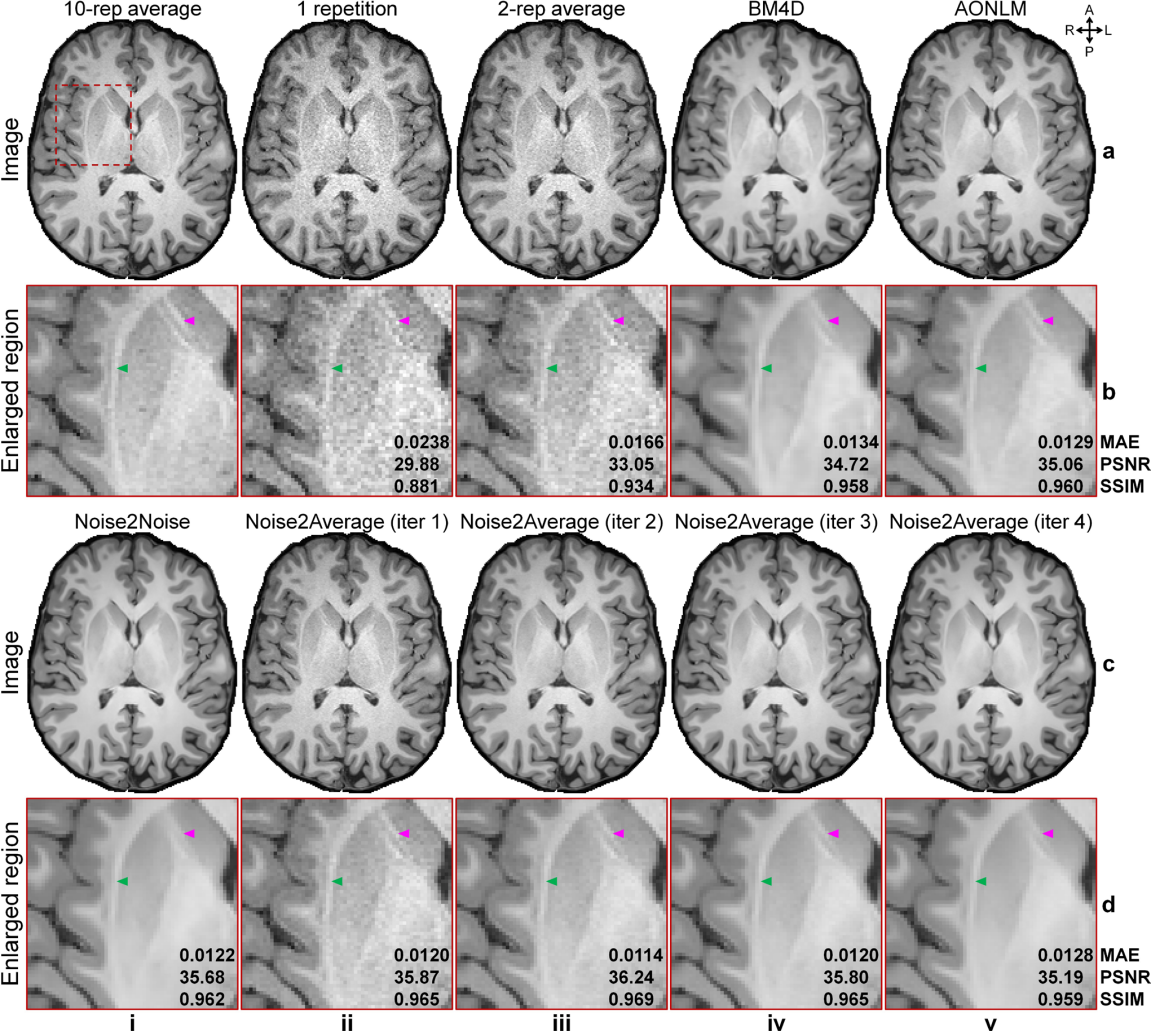
Image results of MGH T1w Wave-MPRAGE data. Exemplary axial image slices from the 10-repetition averaged image volume (ground truth, a, i), single noisy image volume (a, ii), two-repetition averaged image volume (a, iii), BM4D-denoised two-repetition averaged volume (a, iv), AONLM-denoised two-repetition averaged volume (a, v), Noise2Noise-denoised data (c, i), and Noise2Average-denoised data from iterations 1 to 4 (c, ii-v) of a representative subject from MGH T1w Wave-MPRAGE data are shown, along with enlarged views (b, d) of a region of interest (red box in a, i). The arrowheads highlight the claustrum (green) and caudolenticular gray bridges (magenta) with fine textures. Mean absolute error (MAE), peak SNR (PSNR), and structural similarity index (SSIM) are listed to quantify the similarity between different images and the ground truth.

Noise2Average outperformed other methods in denoising empirically acquired and co-registered two repetitions of T1w Wave-MPRAGE data (Fig. 5, Table 1). After each iteration, Noise2Average results gradually became cleaner with stronger denoising effects, at the cost of becoming smoother (Fig. 5c, d, ii-v). The denoised image from the second iteration of Noise2Average (Fig. 5c, d, iii) appeared natural and similar to the ground-truth image obtained by averaging 10 repetitions of the data (Fig. 5a, b, i), with preserved image sharpness and fine textures (e.g., around the claustrum and caudolenticular gray bridges highlighted by the green and magenta arrowheads in Fig. 5b, d). Resultant images from BM4D (Fig. 5a, b, iv), AONLM (Fig. 5a, b, v), Noise2Noise (Fig. 5c, d, i), and Noise2Average at iteration 4 (Fig. 5c, d, v) appeared slightly blurry and lacked fidelity compared with the ground truth (Fig. 5a, b, i). The fine textures around the claustrum and caudolenticular gray bridges in the Noise2Noise results were smoothed out (Fig. 5c, d, i arrowheads).

**Table 1.** Image metrics of MGH T1w Wave-MPRAGE data.

	-	a	b	c	d
	-	1 rep	2-rep avg	BM4D	AONLM
MAE (×10^-2^)	-	2.91 ± 0.259	2.04 ± 0.203	1.65 ± 0.184	1.61 ± 0.181
PSNR (dB)	-	28.15 ± 0.825	31.28 ± 0.897	32.90 ± 0.995	33.10 ± 1.03
SSIM	-	0.850 ± 0.0172	0.915 ± 0.0112	0.942 ± 0.0104	0.944 ± 0.0105

	e	f	g	h	i
	Noise2Noise	N2A (iter 1)	N2A (iter 2)	N2A (iter 3)	N2A (iter 4)
MAE (×10^-2^)	1.51 ± 0.162	1.49 ± 0.158	**1.40 ± 0.155**	1.47 ± 0.160	1.56 ± 0.165
PSNR (dB)	33.78 ± 0.928	33.96 ± 0.931	**34.45 ± 0.969**	34.02 ± 0.955	33.48 ± 0.928
SSIM	0.948 ± 0.0089	0.953 ± 0.0072	**0.958 ± 0.0073**	0.952 ± 0.0085	0.944 ± 0.0095

The group means (± group standard deviations) of the mean absolute error (MAE), peak signal-to-noise ratio (PSNR), and structural similarity index (SSIM) between the single noisy image volume (a), 2-repetition averaged image volume (b), BM4D-denoised 2-repetition averaged volume (c), AONLM-denoised 2-repetition averaged volume (d), Noise2Noise-denoised data (e), and Noise2Average (N2A)-denoised data from iterations 1 to 4 (f-i) and the ground truth of 10 evaluation subjects from MGH T1w Wave-MPRAGE data are listed. Bold and underlined values indicate the best and the second-best performance, respectively. Note that all iterations of N2A are grouped as a single method for ranking purpose.

Quantitatively, the group means (± group standard deviations) of the MAE, PSNR, SSIM compared with the ground truth across 10 evaluation subjects from MGH T1w Wave-MPRAGE data are listed in Table 1. Noise2Average at the second iteration achieved the lowest MAE and the highest PSNR and SSIM (Table 1g) among all denoising methods and different iterations of Noise2Average, consistent with visual inspection. Noise2Noise achieved the second-best performance (Table 1e), superior to the conventional denoising algorithm BM4D (Table 1c) and AONLM (Table 1d). Noise2Average at all iterations (Table 1f-i) outperformed BM4D (Table 1c) and AONLM (Table 1d), while Noise2Average from iterations 1 to 3 (Table 1f-h) outperformed Noise2Noise (Table 1e). BM4D and AONLM results were similar.

**Fig. 6.**
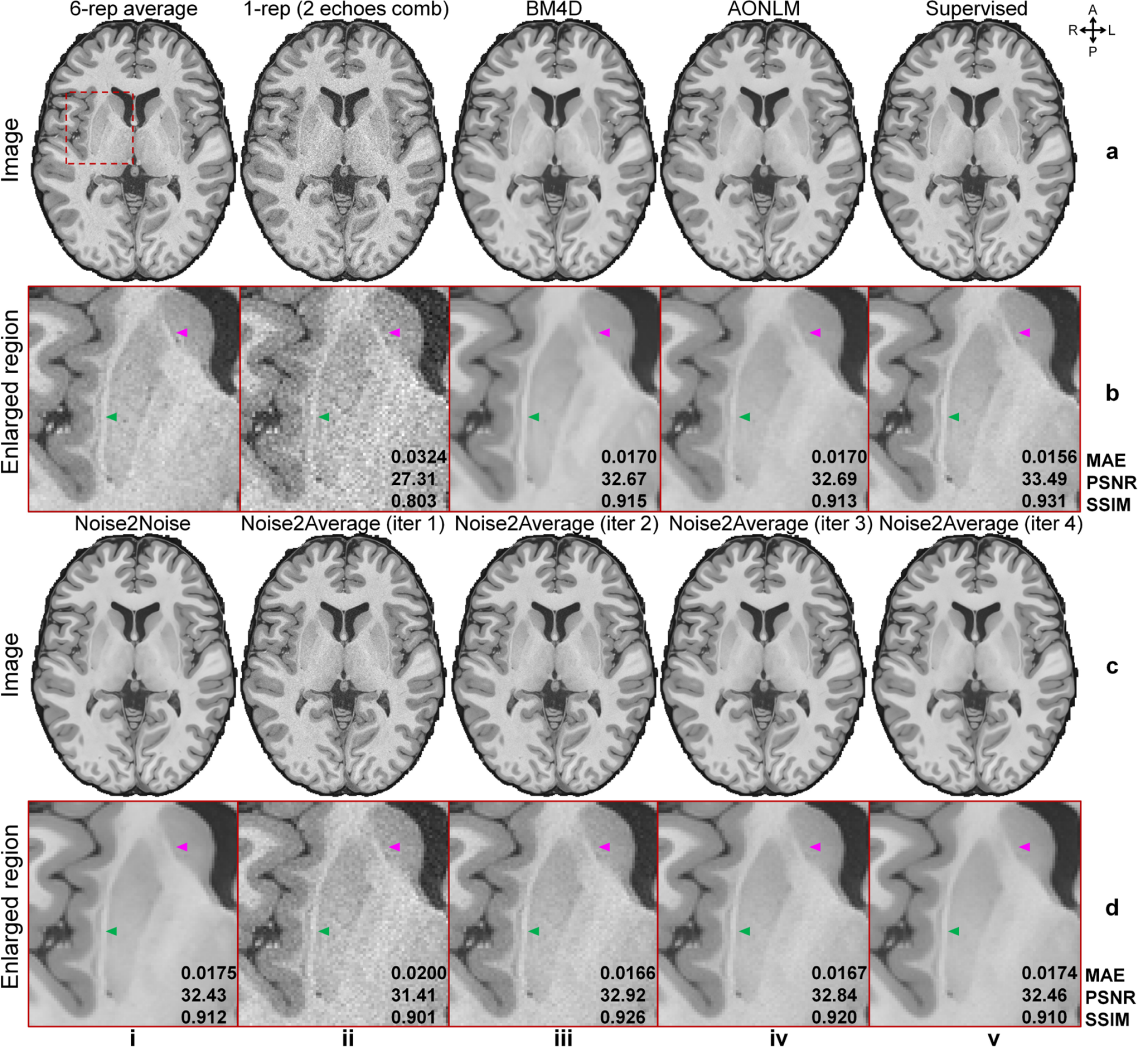
Image results of MGH T1w ME-MPRAGE data. Exemplary axial image slices from the six-repetition averaged image volume (ground truth, a, i), single noisy image volume that is the root mean square of the two images with different echo times (a, ii), BM4D-denoised data (a, iii), AONLM-denoised data (a, iv), supervised learning-denoised data (a, v), Noise2Noise-denoised data (c, i), and Noise2Average-denoised data from iterations 1 to 4 (c, ii-v) of a representative subject from MGH T1w ME-MPRAGE data are shown, along with enlarged views (rows b, d) of a region of interest (the red box in a, i). The arrowheads highlight the claustrum (green) and caudolenticular gray bridges (magenta) with fine textures. Mean absolute error (MAE), peak SNR (PSNR), and structural similarity index (SSIM) are listed to quantify the similarity between different images and the ground truth.

Noise2Average effectively removed noise from the MGH T1w ME-MPRAGE data (Fig. 6 and Table 2), despite the contrast differences between the images from two echo times (Fig. 2e, f). The resultant images gradually became cleaner but smoother at each iteration (Fig. 6c, d, ii-v). The denoised image from the second iteration of Noise2Average (Fig. 6c, d, iii) appeared natural and most similar to the resultant image from the supervised denoising and ground-truth image obtained by averaging six repetitions, with fine textures preserved (Fig. 6c, d, iii, arrowheads). The caudolenticular gray bridge in the Noise2Noise results was smoothed out (Fig. 6c, d, i, magenta arrowhead). Resultant images from BM4D (Fig. 6a, b, iii), AONLM (Fig. 6a, b, iv), Noise2Noise (Fig. 6c, d, i), and Noise2Average at iteration 4 (Fig. 6c, d, v) appeared slightly blurry and unrealistic compared with the ground truth (Fig. 6a, b, i).

**Table 2.** Image metrics of MGH T1w ME-MPRAGE data.

	-	a	b	c	d
	-	1 repetition (2 echoes)	BM4D	AONLM	Supervised
MAE (×10^-2^)	-	3.49 ± 0.371	1.85 ± 0.193	1.84 ± 0.191	**1.68 ± 0.191**
PSNR (dB)	-	26.80 ± 0.909	32.15 ± 0.792	32.17 ± 0.782	**33.02 ± 0.889**
SSIM	-	0.801 ± 0.0256	0.908 ± 0.0156	0.907 ± 0.0145	**0.927 ± 0.0132**

	e	f	g	h	i
	Noise2Noise	N2A (iter 1)	N2A (iter 2)	N2A (iter 3)	N2A (iter 4)
MAE (×10^-2^)	1.92 ± 0.226	2.12 ± 0.225	1.79 ± 0.209	1.84 ± 0.224	1.95 ± 0.250
PSNR (dB)	31.66 ± 0.891	31.06 ± 0.855	32.41 ± 0.869	32.11 ± 0.903	31.57 ± 0.945
SSIM	0.904 ± 0.0190	0.899 ± 0.0163	0.921 ± 0.0145	0.913 ± 0.0170	0.900 ± 0.0200

The group means (± group standard deviations) of the mean absolute error (MAE), peak signal-to-noise ratio (PSNR), and structural similarity index (SSIM) between the single noisy image volume that is the root mean square of the two image volumes with different echo times (a), BM4D-denoised data (b), AONLM-denoised data (c), supervised learning-denoised data (d), Noise2Noise-denoised data (e), and Noise2Average (N2A)-denoised data from iterations 1 to 4 (f-i) and the ground truth of five evaluation subjects from MGH T1w ME-MPRAGE data are listed. Bold and underlined values indicate the best and the second-best performance, respectively.

The group means (± group standard deviations) of the MAE, PSNR, SSIM compared with the ground truth across five evaluation subjects from MGH T1w ME-MPRAGE data are listed in Table 2. Supervised denoising achieved the lowest MAE and highest PSNR and SSIM (Table 2d), as expected. Noise2Average at the second iteration achieved the second-best performance (Table 2g). Unlike results for MGH T1w Wave-MPRAGE data, the performance of Noise2Noise (Table 2e) was inferior to that of conventional method BM4D (Table 2b) and AONLM (Table 2c), presumably suffering from the image contrast difference between images with different echo times. BM4D and AONLM results were similar.

**Fig. 7.**
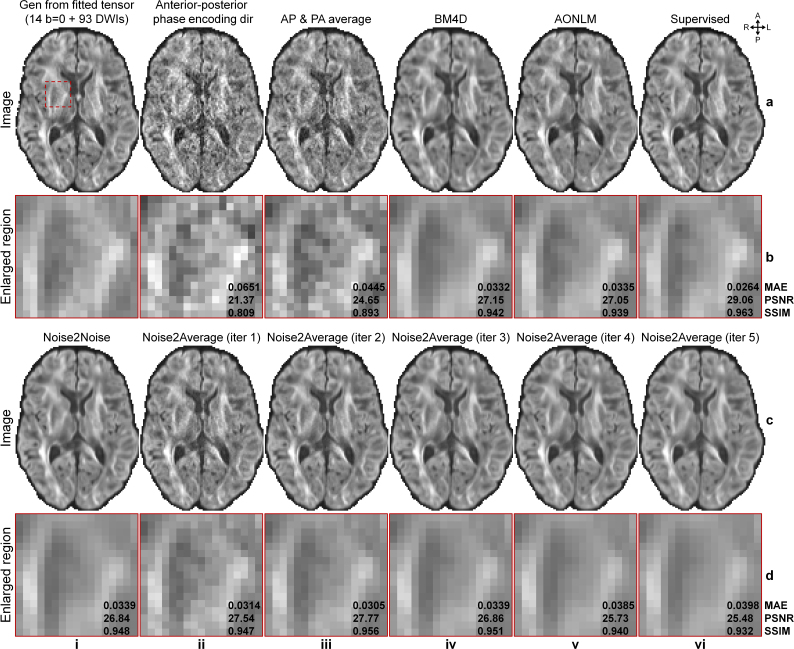
Image results of HCP-A diffusion data. Exemplary axial image slices of the diffusion-weighted image (DWI) volume (along diffusion-encoding direction [-0.85, -0.15, -0.50]) synthesized from ground-truth tensors (a, i), DWI volume acquired with anterior–posterior (AP) phase encoding direction (a, ii), the average of DWI volumes acquired with AP and PA phase encoding directions (a, iii), BM4D-denoised averaged volume (a, iv), AONLM-denoised averaged volume (a, v), supervised learning-denoised averaged volume (a, vi), Noise2Noise-denoised data (c, i), Noise2Average-denoised data from iterations 1 to 5 (c, ii-vi) of a representative subject of HCP-A diffusion data are displayed, along with enlarged views (b, d) of a region of interest near the internal capsule (red box in a, i). Mean absolute error (MAE), peak SNR (PSNR), and structural similarity index (SSIM) are listed to quantify the similarity between different images and the ground truth.

Noise2Average was also effective in denoising co-registered image volumes acquired with opposite phase encoding directions from HCP-A diffusion data (Fig. 7, Table 3), despite the geometric misalignment in regions near the air-tissue interface with severe susceptibility-induced image artifacts remaining after FSL’s “topup” correction (Fig. 3d, iii). Consistent with results from T1w data, Noise2Average gradually became cleaner but smoother at each iteration (Fig. 7c, d, ii-vi). Supervised denoising (Fig. 7a, b, vi) and Noise2Average (Fig. 7c, d, ii, iii) results preserved more textural details and appeared more similar to the ground truth (Fig. 7a, b, i), while results from BM4D (Fig. 7a, b, iv), AONLM (Fig. 7a, b, v), and Noise2Noise (Fig. 7c, d, i) were over smoothed.

**Table 3.** Image metrics of HCP-A diffusion data.

	-	a	b	c	d	e
	-	AP	AP & PA avg	BM4D	AONLM	Supervised
MAE (×10^-2^)	-	6.08 ± 0.711	4.02 ± 0.490	3.11 ± 0.363	3.12 ± 0.366	**2.34 ± 0.327**
PSNR (dB)	-	21.87 ± 0.939	25.38 ± 0.907	27.46 ± 0.813	27.43 ± 0.824	**29.65 ± 0.993**
SSIM	-	0.828 ± 0.0307	0.910 ± 0.0187	0.948 ± 0.0103	0.947 ± 0.0107	**0.970 ± 0.0066**

	f	g	h	i	j	k
	Noise2Noise	N2A (iter 1)	N2A (iter 2)	N2A (iter 3)	N2A (iter 4)	N2A (iter 5)
MAE (×10^-2^)	3.24 ± 0.395	2.90 ± 0.357	2.90 ± 0.358	3.19 ± 0.377	3.54 ± 0.426	3.82 ± 0.406
PSNR (dB)	27.03 ± 0.901	27.92 ± 0.886	27.86 ± 0.905	27.12 ± 0.898	26.27 ± 0.929	25.65 ± 0.877
SSIM	0.952 ± 0.0095	0.954 ± 0.0096	0.960 ± 0.0079	0.954 ± 0.0088	0.945 ± 0.0100	0.936 ± 0.0117

The group means (± group standard deviations) of mean absolute error (MAE), peak signal-to-noise ratio (PSNR), and structural similarity index (SSIM) between the diffusion-weighted image (DWI) volume (along diffusion-encoding direction [-0.85, -0.15, -0.50]) acquired with anterior-posterior (AP) phase encoding direction (a), the average of DWI volumes acquired with AP and PA phase encoding directions (b), BM4D-denoised averaged volume (c), AONLM-denoised averaged volume (d), supervised learning-denoised averaged volume (e), Noise2Noise-denoised data (f), Noise2Average (N2A)-denoised data from iterations 1 to 5 (g-k), and the ground truth of 10 evaluation subjects from HCP-A diffusion data are listed. Bold and underlined values indicate the best and the second-best performance, respectively.

Quantitatively, the group means (± group standard deviations) of the MAE, PSNR, SSIM compared with the ground truth across 10 evaluation subjects from HCP-A diffusion data are listed in Table 3. The supervised denoising achieved the lowest MAE and highest PSNR and SSIM (Table 3e), as expected. The metrics of Noise2Average at the first and second iteration were very similar (Table 3g vs. h), performing the second best. Noise2Noise (Table 3f) did not outperform BM4D (Table 3c) and AONLM (Table 3d) in terms of MAE and PSNR and vice versa for SSIM. BM4D and AONLM results were similar. Noise2Average from iterations 1 to 3 (Table 3g-i) outperformed Noise2Noise (Table 3f).

**Fig. 8.**
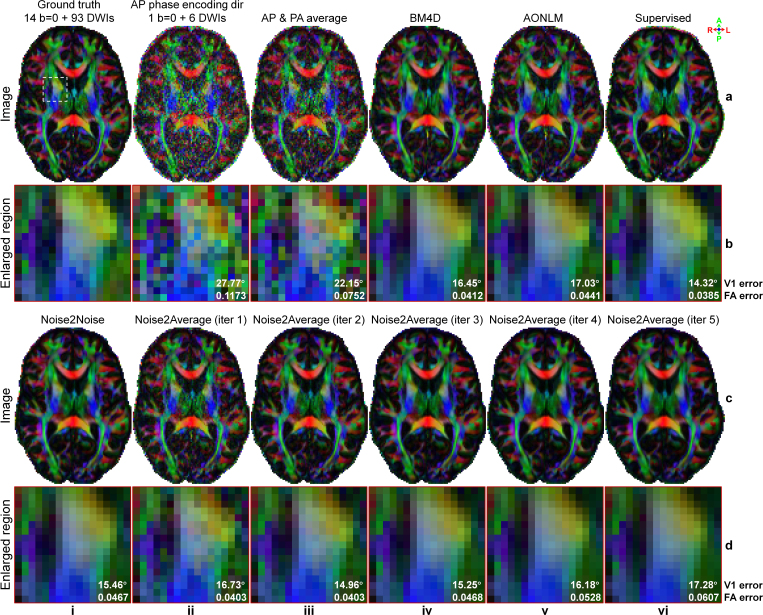
DTI maps of HCP-A diffusion data. Exemplary axial maps of primary eigenvector (V1) encoded fractional anisotropy (FA) volume (red: left-right; green: anterior-posterior; blue: superior-inferior) derived from 14 b = 0 image volumes and 93 diffusion-weighted image (DWI) volumes (each is the average of 2 volumes from anterior-posterior (AP) and PA phase encoding directions, ground truth) (a, i), 1 b = 0 and 6 DWI volumes acquired along AP phase encoding direction (a, ii), 1 b = 0 and 6 DWI volumes (AP and PA averaged) (a, iii), BM4D-denoised data (a, iv), AONLM-denoised data (a, v), supervised learning-denoised data (a, vi), Noise2Noise-denoised data (c, i), and Noise2Average-denoised data from iterations 1 to 5 (c, ii-vi) of a representative subject of HCP-A diffusion data are displayed, along with enlarged views (b, d) of a region of interest near the internal capsule (white box in a, i). The mean absolute errors within the gray matter and white matter are listed to quantify the similarity between V1 and FA from different images and the ground truth.

In addition to improving image quality, Noise2Average also increased the accuracy of quantitative metrics from DTI using image volume pairs acquired with opposite phase encoding directions from HCP-A diffusion data (Fig. 8, Table 4). Resultant V1 encoded FA maps from Noise2Average also gradually became cleaner but smoother (Fig. 8c, d, ii-vi). The V1 encoded FA maps from Noise2Average iterations 1 (Fig. 8c, d, ii) and 2 (Fig. 8c, d, iii) appeared similar to the ground-truth map (Fig. 8a, b, i) obtained using ~15.3× more data (Fig. 8a, b, ii) and supervised learning denoised map (Fig. 8a, b, vi), with more textural details retained compared with those from BM4D, AONLM and Noise2Noise.

**Table 4.** DTI metrics of HCP-A diffusion data.

Mean absolute	-	a	b	c	d	e
Error	-	AP	AP & PA average	BM4D	AONLM	Supervised
V1 error (°)	-	28.735 ± 2.396	22.788 ± 2.173	17.149 ± 1.836	17.794 ± 1.808	**14.661 ± 2.043**
FA error	-	0.121 ± 0.017	0.0748 ± 0.00963	0.0412 ± 0.00373	0.0442 ± 0.00412	**0.0346 ± 0.0037**
MD error (μm^2^/ms)	-	0.0673 ± 0.0114	0.0374 ± 0.00582	0.0357 ± 0.00627	0.0333 ± 0.00527	**0.0277 ± 0.00525**
AD error (μm^2^/ms)	-	0.148 ± 0.0266	0.0851 ± 0.0137	0.0589 ± 0.00766	0.0589 ± 0.00687	**0.0461 ± 0.00458**
RD error (μm^2^/ms)	-	0.0784 ± 0.0105	0.0466 ± 0.00527	0.0391 ± 0.0057	0.0378 ± 0.00534	**0.0310 ± 0.00609**

Mean absolute	f	g	h	i	j	k
Error	Noise2Noise	N2A (iter 1)	N2A (iter 2)	N2A (iter 3)	N2A (iter 4)	N2A (iter 5)
V1 error (°)	16.319 ± 1.910	17.460 ± 2.045	15.783 ± 2.065	16.228 ± 2.302	17.346 ± 2.622	18.479 ± 2.767
FA error	0.0463 ± 0.00401	0.0394 ± 0.00358	0.0400 ± 0.00342	0.0474 ± 0.00462	0.0538 ± 0.0046	0.0591 ± 0.00483
MD error (μm^2^/ms)	0.0357 ± 0.00562	0.0293 ± 0.00491	0.0317 ± 0.00546	0.0355 ± 0.00614	0.0391 ± 0.0066	0.0423 ± 0.00709
AD error (μm^2^/ms)	0.0605 ± 0.00437	0.0520 ± 0.0058	0.0526 ± 0.00511	0.0587 ± 0.00613	0.0644 ± 0.00741	0.0694 ± 0.0085
RD error (μm^2^/ms)	0.0401 ± 0.00663	0.0336 ± 0.00492	0.0364 ± 0.00601	0.0418 ± 0.00682	0.0469 ± 0.00681	0.0512 ± 0.00682

The group means (± group standard deviations) of the mean absolute errors between DTI metrics, including primary eigenvector (V1), fractional anisotropy (FA), mean diffusivity (MD), axial diffusivity (AD), and radial diffusivity (RD), derived from one b = 0 and six DWI volumes acquired along anterior–posterior (AP) phase encoding direction (a), one b = 0 and six DWI volumes (AP and PA averaged) (b), BM4D-denoised data (c), AONLM-denoised data (d), supervised learning-denoised data (e), Noise2Noise-denoised data (f), and Noise2Average (N2A)-denoised data from iterations 1 to 5 (g-k) and the ground truth of 10 evaluation subjects from HCP-A diffusion data. Bold and underlined values indicate the best and the second-best performance, respectively.

The group means (± group standard deviations) of the MAE for five DTI metrics, including V1, FA, MD, AD, and RD, compared with the ground truth across 10 evaluation subjects from HCP-A diffusion data are listed in Table 4. Supervised denoising outperformed all other methods for all five DTI metrics, as expected. Partially consistent with results for image quality, Noise2Average at iteration 1 (Table 4g) achieved the second lowest MAEs in terms of scalar DTI metrics (FA, MD, AD, and RD), while Noise2Average at iteration 2 (Table 4h) achieved the second lowest MAE for orientation DTI metric V1. Notably, Noise2Average consistently outperformed Noise2Noise at iteration 2, achieving MAE decreases of 3.2%, 13.6%, 11.2%, 13.1%, and 9.2% compared with Noise2Noise for V1, FA, MD, AD, and RD, respectively. Noise2Noise (Table 4f) outperformed BM4D (Table 4c) and AONLM (Table 4d) in terms of V1 but generated less accurate scalar metrics including FA, MD, AD, and RD. Despite similar results for image quality, the performance of BM4D and AONLM for DTI metrics was more variable.

**Fig. 9.**
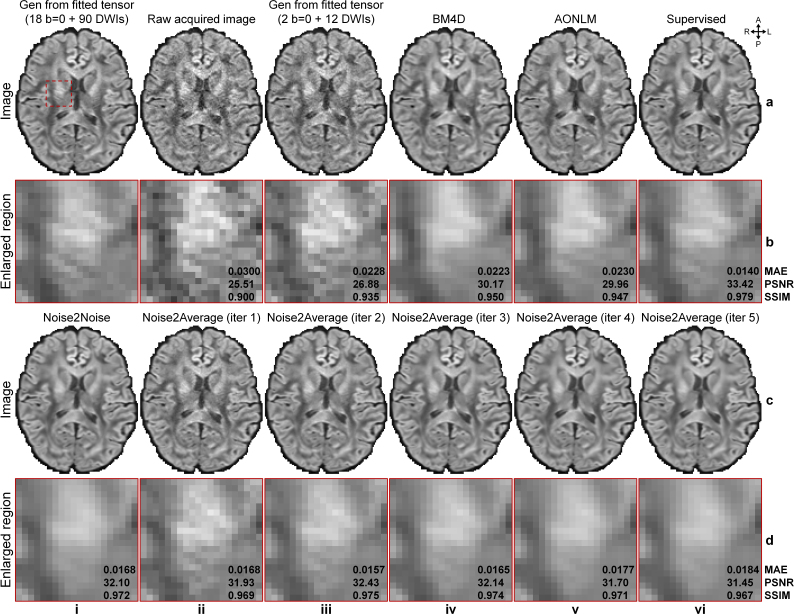
Image results of WU-Minn-Ox HCP diffusion data. Exemplary axial image slices of the diffusion-weighted image (DWI) volume (along diffusion-encoding direction [-0.14, 0.49, -0.86]) synthesized from ground-truth tensors (a, i, ground truth), raw acquired image volume (a, ii), DWI volume synthesized from tensors fitted using 2 b = 0 and 12 DWI volumes (a, iii), BM4D-denoised raw DWI volume (a, iv), AONLM-denoised raw DWI volume (a, v), supervised learning-denoised raw DWI volume (a, vi), Noise2Noise-denoised data (c, i), Noise2Average-denoised data from iterations 1 to 5 (c, ii-vi) of a representative subject from WU-Minn-Ox HCP diffusion data are displayed, along with enlarged views (b, d) of a region of interest near the internal capsule (red box in a, i). Noise2Noise and Noise2Average results were the average of two denoised repetitions of image volumes. Mean absolute error (MAE), peak SNR (PSNR), and structural similarity index (SSIM) are listed to quantify the similarity between different images and the ground truth.

Noise2Average also successfully removed noise using two repetitions of DWI volumes synthesized using the diffusion tensor model from WU-Minn-Ox HCP diffusion data (Fig. 9, Table 5). Consistent with other results, Noise2Average results appeared cleaner with stronger denoising effects but got smoother after each iteration (Fig. 9c, d, ii-vi). Supervised denoising (Fig. 9a, b, vi) and the first two iterations of Noise2Average (Fig. 9c, d, ii, iii) results contained more textural details (e.g., near the internal capsule in Fig. 9b, d) and appeared more similar to the ground truth (Fig. 9a, b, i). BM4D (Fig. 9a, b, iv) and Noise2Noise (Fig. 9c, d, i) results were over smoothed with all textural details in the internal capsule lost (Fig. 9b, d). AONLM (Fig. 9a, b, v) induced less blurring compared with BM4D and Noise2Noise. BM4D and AONLM results were from a single raw DWI volume and therefore exhibited slightly lower SNR compared with those from Noise2Noise and Noise2Average, which were average of two repetitions of denoised image volumes.

**Table 5.** Image metrics of WU-Minn-Ox HCP diffusion data.

	a	b	c	d	e	f
	Raw	Synthesized	BM4D	AONLM	Supervised	MPPCA
MAE (×10^-2^)	3.27 ± 0.167	2.52 ± 0.142	2.48 ± 0.155	2.53 ± 0.142	**1.61 ± 0.123**	2.56 ± 0.097
PSNR (dB)	24.22 ± 0.756	25.42 ± 0.872	28.82 ± 0.741	28.68 ± 0.715	**31.53 ± 1.215**	28.47 ± 0.55
SSIM	0.886 ± 0.0114	0.924 ± 0.0089	0.941 ± 0.0072	0.938 ± 0.0068	**0.975 ± 0.0032**	0.919 ± 0.0052

	g	h	i	j	k	l
	LCPCA	Patch2Self	Noise2Noise	N2A (iter 1)	N2A (iter 2)	N2A (iter 3)
MAE (×10^-2^)	1.90 ± 0.086	2.53 ± 0.127	1.86 ± 0.128	1.92 ± 0.146	1.77 ± 0.137	1.83 ± 0.133
PSNR (dB)	26.11 ± 0.89	25.39 ± 0.73	30.59 ± 1.063	30.25 ± 1.012	30.80 ± 1.115	30.67 ± 1.092
SSIM	0.952 ± 0.0030	0.922 ± 0.0058	0.968 ± 0.0035	0.962 ± 0.0052	0.971 ± 0.0038	0.970 ± 0.0036

The group means (± group standard deviations) of mean absolute error (MAE), peak signal-to-noise ratio (PSNR), and structural similarity index (SSIM) between the raw acquired diffusion-weighted image (DWI) volume shown in Figure 9a, ii (a), DWI volume synthesized from tensors fitted using 2 b = 0 and 12 DWI volumes (b), BM4D-denoised raw DWI volume (c), AONLM-denoised raw DWI volume (d), supervised learning-denoised data (e), MPPCA-denoised data (f), LCPCA-denoised data (g), Patch2Self-denoised data (h), Noise2Noise-denoised data (i), Noise2Average (N2A)-denoised data from iterations 1 to 3 (j-l) approximately along the superior–inferior direction (i.e., [-0.18, 0.26, -0.95]) and the ground truth of 10 evaluation subjects from WU-Minn-Ox HCP diffusion data. Bold and underlined values indicate the best and the second-best performance, respectively.

Quantitatively, the group means (± group standard deviations) of the MAE, PSNR, SSIM compared with the ground truth across 10 evaluation subjects from WU-Minn-Ox HCP diffusion data are listed in Table 5. Noise2Average at iteration 2 achieved the second-best performance (Table 5k), which was comparable with that of supervised denoising (Table 5e). BM4D and AONLM results were from a single raw DWI volume and, therefore, resulted in lower similarity metrics then those from Noise2Noise and Noise2Average results (Table 5c, d vs. Table 5i-l), which, however, substantially improved upon those from raw data (Table 5a). Compared with methods tailored for diffusion MRI, including MPPCA, LCPCA, and Patch2Self, both Noise2Average and Noise2Noise demonstrated superior improvements in image quality (Table 5f-h vs. Table 5i-l). Noise2Average at iterations 2 to 3 (Table 5k-l) outperformed Noise2Noise (Table 5i).

**Fig. 10.**
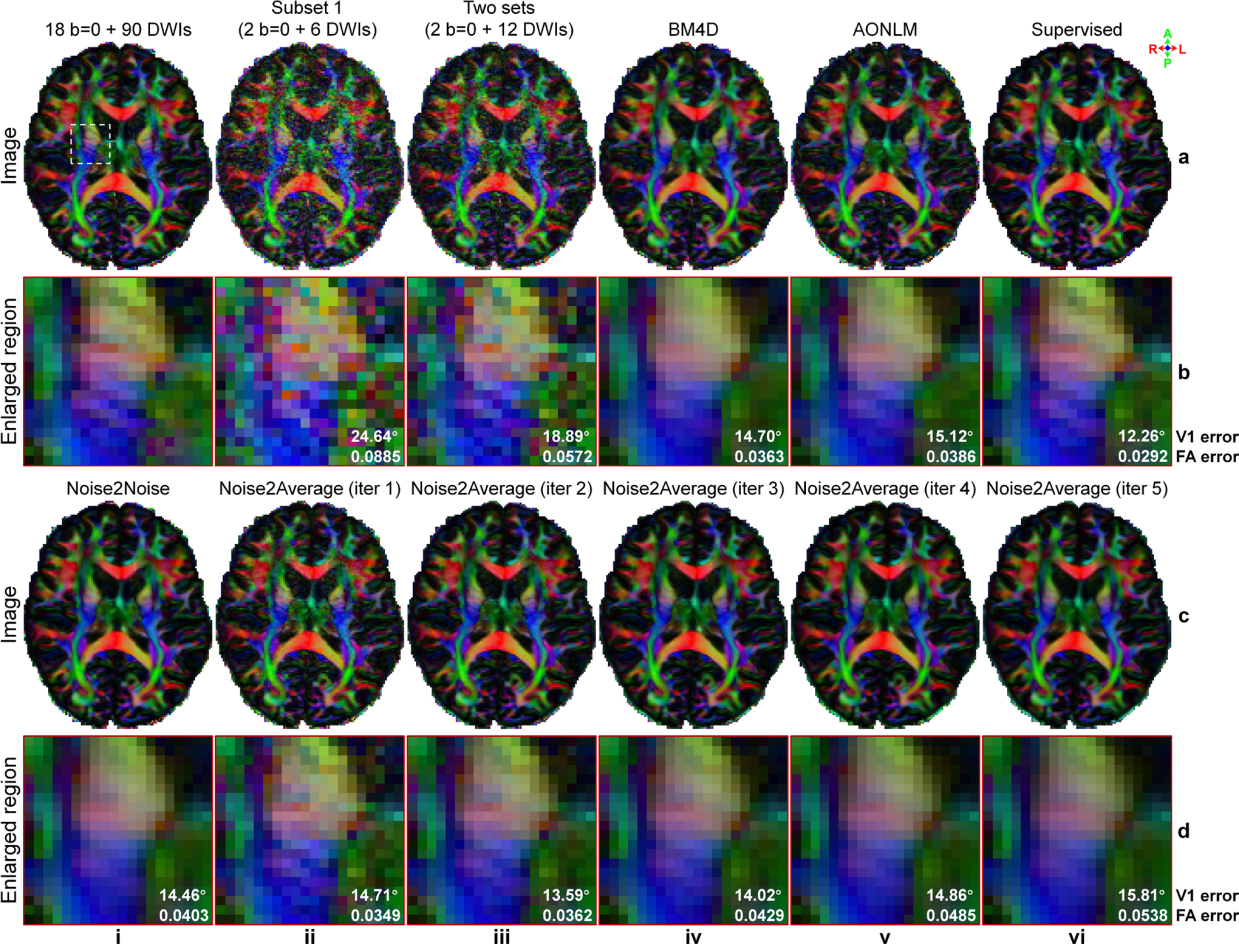
DTI maps of WU-Minn-Ox HCP diffusion data. Exemplary axial maps of primary eigenvector (V1) encoded fractional anisotropy (FA) volume (red: left-right; green: anterior-posterior; blue: superior-inferior) derived from 18 b = 0 image volumes and 90 diffusion-weighted image (DWI) volumes (a, i, ground truth), 2 b = 0 and 6 DWI volumes (a, ii), 2 b = 0 and 12 DWI volumes (a, iii), BM4D-denoised data (a, iv), AONLM-denoised data (a, v), supervised learning-denoised data (a, vi), Noise2Noise-denoised data (c, i), and Noise2Average-denoised data from iterations 1 to 5 (c, ii-vi) of a representative subject from WU-Minn-Ox HCP diffusion data are displayed, along with enlarged views (b, d) of a region of interest near the internal capsule (white box in a, i). The mean absolute errors within the gray matter and white matter are listed to quantify the similarity between V1 and FA from different images and the ground truth.

Noise2Average improved not only image quality but also the accuracy of DTI metrics using two repetitions of synthesized DWI volumes from WU-Minn-Ox HCP diffusion data (Fig. 10, Table 6). The V1 encoded FA maps from Noise2Average iterations 1 (Fig. 10c, d, ii) and 2 (Fig. 10c, d, iii) appeared similar to the ground-truth map (Fig. 10a, b, i) obtained using ~7.7× more data and the supervised learning denoised map (Fig. 10a, b, vi), with more textural details retained. Therefore, their V1 encoded FA maps displayed with exquisite detail the characteristic stripes seen in the internal capsule (i.e., the gray matter bridges, Fig. 10b, d), which were contaminated by noise in the map derived from raw data (Fig. 10b, iii) and blurred out in the map derived from BM4D- (Fig. 10b, iv), AONLM- (Fig. 10b, v), and Noise2Noise-denoised data (Fig. 10d, i) as well as Noise2Average-denoised data using more than three iterations (Fig. 10d, iv-vi). AONLM preserved slightly more textural details than BM4D and Noise2Noise.

**Table 6.** DTI metrics of WU-Minn-Ox HCP diffusion data.

Mean absolute	a	b	c	d	e	f
Error	Subset 1 (2 b = 0 + 6 DWIs)	Two sets (2 b = 0 + 12 DWIs)	BM4D	AONLM	Supervised	MPPCA
V1 error (°)	26.383 ± 1.166	20.510 ± 1.080	16.176 ± 1.083	16.566 ± 1.029	**13.256 ± 0.819**	20.0367 ± 1.228
FA error	0.0993 ± 0.00772	0.0648 ± 0.00531	0.0388 ± 0.0028	0.0412 ± 0.00293	**0.0318 ± 0.00187**	0.0542 ± 0.00353
MD error (μm^2^/ms)	0.0475 ± 0.00329	0.0414 ± 0.00346	0.0405 ± 0.00282	0.0424 ± 0.00435	**0.0323 ± 0.00265**	0.0409 ± 0.00379
AD error (μm^2^/ms)	0.115 ± 0.0079	0.0799 ± 0.00545	0.0609 ± 0.00351	0.0645 ± 0.00447	**0.0483 ± 0.00300**	0.0719 ± 0.00406
RD error (μm^2^/ms)	0.0641 ± 0.00486	0.0497 ± 0.00433	0.0433 ± 0.00291	0.0460 ± 0.00450	**0.0349 ± 0.00256**	0.0471 ± 0.00406

Mean absolute	g	h	i	j	k	l
Error	LCPCA	Patch2Self	Noise2Noise	N2A (iter 1)	N2A (iter 2)	N2A (iter 3)
V1 error (°)	17.731 ± 1.165	25.132 ± 1.044	15.630 ± 1.098	16.086 ± 1.062	14.778 ± 1.010	15.191 ± 0.955
FA error	0.0436 ± 0.00227	0.0684 ± 0.00339	0.0428 ± 0.00313	0.0374 ± 0.00250	0.0374 ± 0.00254	0.0442 ± 0.00324
MD error (μm^2^/ms)	0.0369 ± 0.00346	0.0525 ± 0.00293	0.0407 ± 0.00488	0.0347 ± 0.00292	0.0344 ± 0.00299	0.0360 ± 0.00283
AD error (μm^2^/ms)	0.0623 ± 0.00374	0.0935 ± 0.00337	0.0652 ± 0.00666	0.0547 ± 0.00341	0.0558 ± 0.00434	0.0619 ± 0.00519
RD error (μm^2^/ms)	0.0412 ± 0.00322	0.0583 ± 0.00325	0.0431 ± 0.00351	0.0382 ± 0.00295	0.0372 ± 0.00266	0.0397 ± 0.00278

The group means (± group standard deviations) of the mean absolute errors between DTI metrics, including primary eigenvector (V1), fractional anisotropy (FA), mean diffusivity (MD), axial diffusivity (AD), and radial diffusivity (RD), derived from 2 b = 0 and 6 DWI volumes (a), 2 b = 0 and 12 DWI volumes (b), BM4D-denoised data (c), AONLM-denoised data (d), supervised learning-denoised data (e), MPPCA-denoised data (f), LCPCA-denoised data (g), Patch2Self-denoised data (h), Noise2Noise-denoised data (i), and Noise2Average (N2A)-denoised data from iterations 1 to 3 (j-l) and the ground truth of 10 evaluation subjects from WU-Minn-Ox HCP diffusion data. Bold and underlined values indicate the best and the second-best performance, respectively.

The group means (± group standard deviations) of the MAE for V1, FA, MD, AD, and RD compared with the ground truth across 10 evaluation subjects from HCP WU-Minn-Ox diffusion data are listed in Table 6. Supervised denoising achieved the lowest errors among all the DTI metrics (Table 6e), as expected. Noise2Average at iteration 2 (Table 6k) achieved the second lowest errors for all DTI metrics, except for AD, for which Noise2Average at iteration 1 (Table 6j) achieved the second lowest error. Noise2Noise (Table 6i) produced more accurate V1 and RD while BM4D (Table 6c) and AONLM (Table 6d) produced more accurate scalar metrics including FA, MD, and AD. MPPCA, LCPCA, and Patch2Self exhibited higher MAEs than BM4D and AONLM, potentially due to the very limited number of diffusion-encoding directions used in our experiments (Table 6f-h vs. Table 6c-d). Noise2Average at iterations 1 to 3 outperformed Noise2Noise in terms of all metrics, except for V1 at iteration 1 and FA at iteration 3. Notably, Noise2Average at iteration 2 achieved substantial improvements over Noise2Noise, demonstrating MAE reduction of 5.6%, 12.6%, 15.5%, 14.4%, and 13.7% for V1, FA, MD, AD, and RD, respectively.

The computational requirements for the quantitative experiments are summarized in Table 7. As image resolution increased, the number of extracted blocks grows, leading to longer computation times. For 0.8 mm isotropic resolution T1w images, the denoising process required 14.2 min for two iterations, whereas processing would be faster for the more commonly used 1 mm isotropic resolution data. For diffusion data, GPU memory requirements increased with the number of directions to be denoised. Fortunately, the memory demand remained well below the capacity of most commercial GPUs (typically >12 GB).

**Table 7.** Summary of computational requirements.

Dataset name	Block size	Number blocks	Loss	Epochs per iteration	GPU (GB)	Time per iteration (s)	Time per block (s)
Wave-MPRAGE T1w (0.8 mm iso)	80 × 80 × 80 × 1	108	MSE	10	6.5	427	0.4
MEMPRAGE T1w (0.6 mm iso)	80 × 80 × 80 × 1	384	MSE	10	6.5	1544	0.4
HCP-A Diffusion (1.5 mm iso)	64 × 64 × 64 × 7	48	MAE	20	7.7	716	0.7
WU-Minn-Ox Diffusion (1.25 mm iso)	64 × 64 × 64 × 13	48	MAE	20	8.7	883	0.9

The block size, number of extracted blocks, epochs per iteration, GPU memory requirements for fine-tuning, computation time per fine-tuning iteration, and computation time per block on an NVIDIA RTX 3090 GPU are presented.

**Fig. 11.**
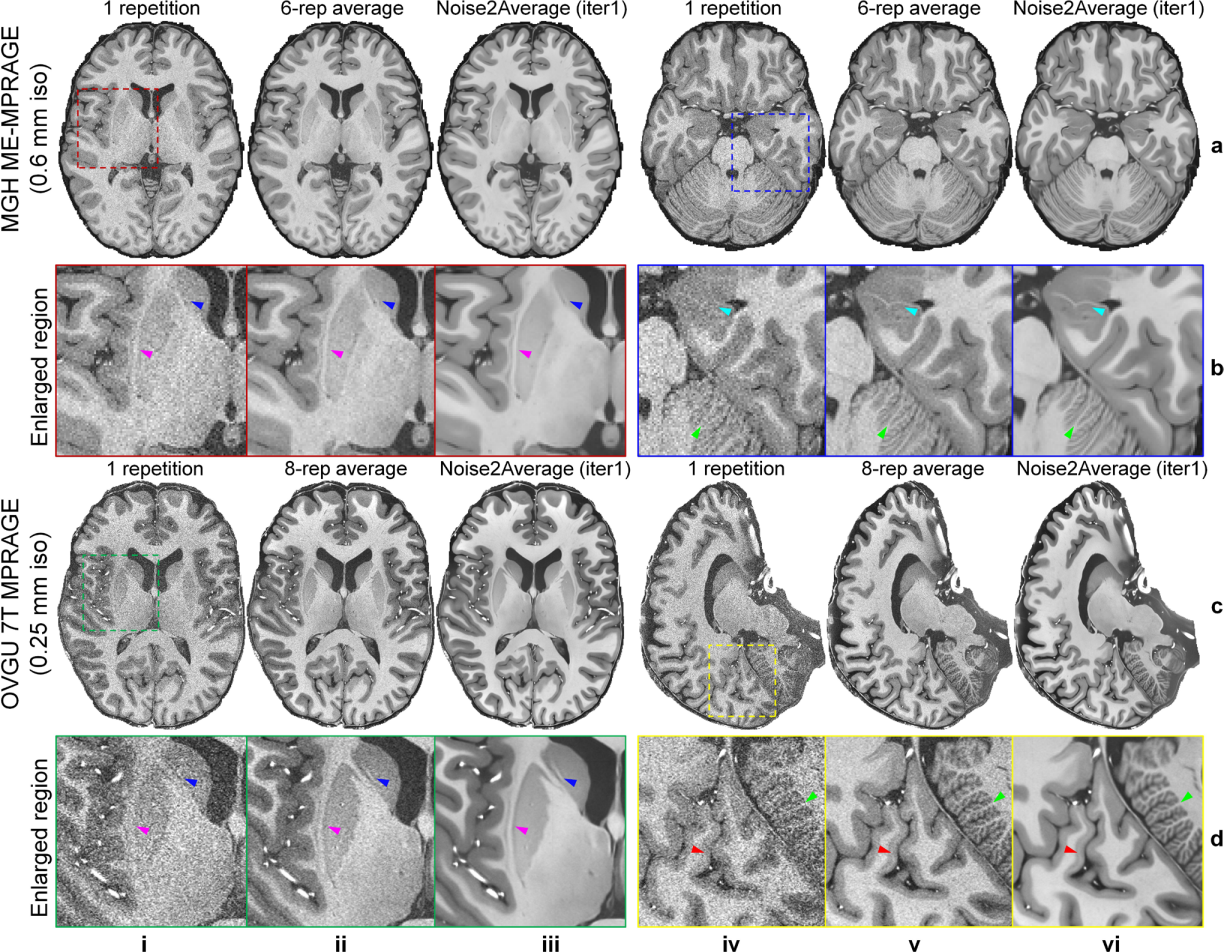
Noise2Average results of ultrahigh resolution T1w data. Exemplary axial and sagittal image slices from the single noisy image volume (rows a, c, columns i, iv), six-repetition or eight-repetition averaged volume (rows a, c, columns ii, v), and Noise2Average-denoised data at iteration 1 (rows a, c, columns iii, vi) of a representative subject from MGH T1w ME-MPRAGE data (a) and OVGU 7T T1w data (c) are displayed, along with enlarged views (b, d) of regions of interest (ROIs, boxes in rows a, c, columns i, iv). Selected ROIs are near the basal ganglia (red box in a, i and green box in c, i), hippocampus (blue box in a, iv), and primary visual cortex (yellow box in c, iv). The arrowheads highlight the claustrum (magenta, b, d, i-iii), caudolenticular gray bridges (blue, b, d, i-iii), hippocampus (cyan, b, iv-vi), cerebellar cortex (green, b, d, iv-vi), and the Stripe of Gennari (red, d, iv-vi).

In addition to denoising data with only two repetitions, Noise2Average easily extended to process data with many repetitions (Fig. 11). At ultrahigh resolution (0.6 mm isotropic and 0.25 mm isotropic for MGH T1w ME-MPRAGE and OVGU 7T T1w data), even the six-repetition (~1-hour scan) and eight-repetition (~7-hour scan) averaged data were still slightly noisy (Fig. 11, ii and v). Noise2Average effectively restored cleaner and less noisy images while preserving sharpness and textural details (Fig. 11, iii and vi) in several regions of interest (ROIs), such as the claustrum (magenta arrowheads, Fig. 11b, d, i-iii), caudolenticular gray bridges (blue arrowheads, Fig. 11b, d, i-iii), hippocampus (cyan arrowheads, Fig. 11b, iv-vi), cerebellar cortex (green arrowheads, Fig. 11b, d, iv-vi), and the Stripe of Gennari (red arrowheads, Fig. 11d, iv-vi). Particularly, the Stripe of Gennari, myelinated fibers that run parallel to the surface of the cerebral cortex that form a white line along the edges of the calcarine sulcus (red arrowhead, Fig. 11d, vi), and folia of the cerebellum, small leaflike laminae (green arrowhead, Fig. 11d, vi) were clearly depicted in Noise2Average-denoised 7T T1w images at 0.25 mm isotropic resolution.

**Fig. 12.**
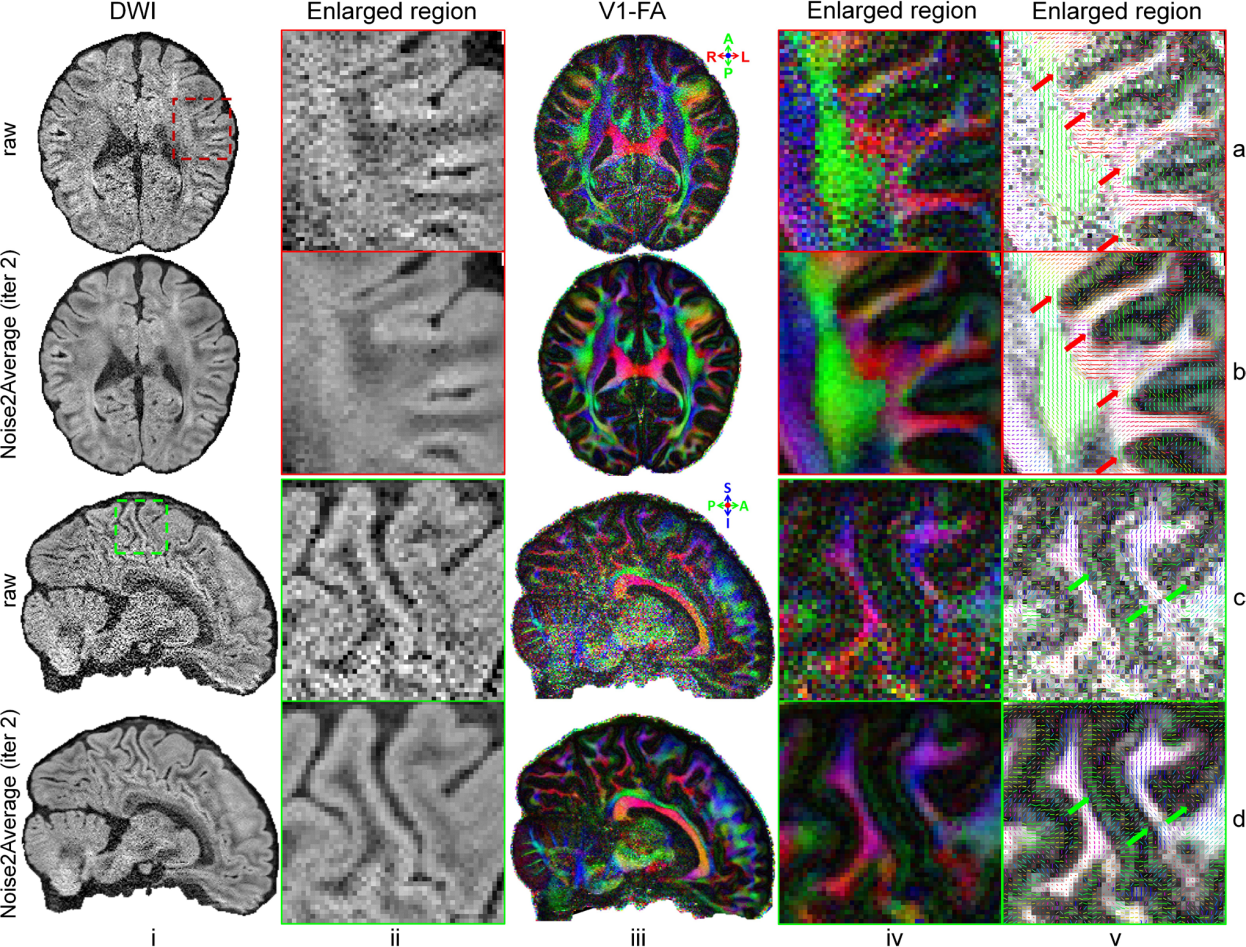
Noise2Average results of ultrahigh resolution diffusion data. Exemplary axial and sagittal image slices from the average of diffusion-weighted image (DWI) volumes acquired with anterior-posterior (AP) and PA phase encoding directions along diffusion-encoding direction [-0.96, -0.06, 0.28] (a, i and c, i) and Noise2Average-denoised image volume at iteration 2 (b, i and d, i) of a representative subject from MGH gSlider-SMS diffusion MRI data are displayed, along with enlarged views (ii) of regions of interest of cortical gyri (red box in a, i and green box in c, i). The DTI primary eigenvector (V1) encoded fractional anisotropy (FA) maps (red: left-right; green: anterior-posterior; blue: superior-inferior) from the raw and Noise2Average-denoised data (iii and iv) are displayed, with V1 rendered as color-encoded sticks superimposed on FA maps (v). The arrowheads highlight U-fibers in the superficial white matter (red, a, b, v) and fiber crossings at the gray-white junction (green, c, d, v).

Noise2Average successfully improved the ultrahigh 0.76 mm isotropic resolution diffusion MRI data using image volume pairs acquired with opposite phase encoding directions, especially in the center of the brain where the sensitivity of receive coils is lower (Fig. 12). In addition to higher image quality (Fig. 12, i, ii), DTI V1 and FA results were also substantially improved (Fig. 12, iii-v). Noise2Average clearly visualized U-fibers connecting cortical regions between adjacent gyri (red arrows, Fig. 12b, v), the clear dark band with reduced FA at the gray–white junction where gray and white matter form fiber crossings (green arrows, Fig. 12d, v), sub-cortical white matter fibers coherently fanning into the cortex (Fig. 12b, d, v), and the coherent fiber orientations in the cortex (i.e., green contours surrounding the gyrus in Fig. 12b, d, iv) that are mostly orthogonal to the cortical surface (Fig. 12b, d, v) in the V1 encoded FA maps from a 18-min DTI scan, which were only roughly preserved in maps from raw data.

## 5 Discussion

In this study, we propose a new learning strategy entitled “Noise2Average” for denoising image data with multiple repetitions to eliminate the requirement for high-SNR reference data for supervising the training. Noise2Average learns to map each noisy repetition to its residual compared with the average of all repetitions, then averages all denoised results to recover higher SNR, and repeats this supervised residual learning-based denoising process for multiple iterations with the denoising result from the previous iteration as the training target. Systematic and quantitative evaluations demonstrate the efficacy of Noise2Average for denoising four different types of empirical T1w and diffusion MRI data including MGH T1w Wave-MPRAGE data (Fig. 5, Table 1), MGH T1w ME-MPRAGE data (Fig. 6, Table 2), HCP-A diffusion data (Figs. 7, 8, Tables 3, 4), and WU-Minn-Ox HCP diffusion data (Figs. 9, 10, Tables 5, 6), as well as MRI data acquired at ultrahigh sub-millimeter resolution including MGH T1w ME-MPRAGE data (Fig. 11a, b), OVGU 7T T1w data (Fig. 11c, d), and MGH gSlider-SMS diffusion MRI data (Fig. 12). Noise2Average not only improves SNR and preserves image sharpness and textural details (Figs. 5, 6, 7, 9, Tables 1, 2, 3, 5), but also increases the accuracy of downstream diffusion MRI signal modeling for mapping human brain microstructure (Figs. 8, 10, Tables 4, 6), which aligns well with the comprehensive evaluation criteria established for diffusion MRI denoising methods (Manzano Patron et al., 2024). The denoising performance of Noise2Average is comparable with that of the supervised learning-based method and superior to that of the classic Noise2Noise method and conventional benchmark methods BM4D and AONLM.

Noise2Average is preferable to Noise2Noise for empirical and simulation data for several reasons. First, the essential assumption of Noise2Noise that the two repetitions of data for denoising only differ in their noise observations is difficult to satisfy in practice. Because of inter-volume and intra-volume motion during the long acquisition (e.g., as long as 97 s for each highly accelerated MGH T1w Wave-MPRAGE volume) and/or distinct imaging parameters (e.g., different echo times for MGH T1w ME-MPRAGE data, different phase-encoding directions for HCP-A diffusion data, and different diffusion-encoding directions for WU-Minn-Ox HCP diffusion data), the two repetitions also differ in geometric correspondence and image intensity especially in regions with spatially and temporally varying image artifacts, which cannot be completely accounted for by image co-registration and artifact correction methods (Figs. 2, 3). As a result, Noise2Noise-denoised images are often blurrier (Figs. 6, 7, 9) and lead to less accurate DTI metrics (Figs. 8, 10, Tables 3, 4). Second, residual learning of Noise2Average allows CNNs to learn only the high-frequency spatial information, which boosts CNN performance, improves CNN generalizability, accelerates convergence, and avoids the vanishing gradient problem, and is, therefore, widely adopted (He et al., 2016; Tian et al., 2020; Tian, Bilgic, et al., 2021; Zhang et al., 2017). Residual learning is particularly helpful in preserving image sharpness and textural details across both empirical data (Fig. 5 and Fig. 6) and simulation data under different noise conditions (Supplementary Figs. S1-S2, Supplementary Table S2). Finally, Noise2Average can easily process more than two noisy repetitions for further boosted denoising performance (Fig. 11).

In synergy with transfer learning, Noise2Average can be self-supervised, which further enhances its practical feasibility. Even though Noise2Average does not require high-SNR reference training data, its CNN still needs to be trained on data from numerous subjects to avoid overfitting, which might be unavailable or is very challenging to acquire for a particular study. For example, the acquisition of two repetitions of OVGU 7T T1w data takes ~100 min. Transfer learning implemented by fine-tuning parameters of pre-trained CNNs not only solves this problem, but also considerably reduces the training time of Noise2Average and renders it even more accessible. Although magnitude MRI data can exhibit Rician or non-central Chi distributions and contrast variations due to differing hardware and acquisition protocols, we hypothesized that the pre-training stage, which primarily initializes the network parameters, remains robust to such variations (Supplementary Fig. S3). Denoising results obtained with models pre-trained on simulated Gaussian noise versus Rician noise were closely matched both visually and quantitatively, indicating that the characteristics of the pre-training data need not perfectly match those of the target denoising data. Moreover, Noise2Average models pre-trained on open-access T1w and diffusion data from the large-scale HCP were effective across several acquisition protocols in our study, further demonstrating the practical feasibility of Noise2Average.

**Fig. 13.**
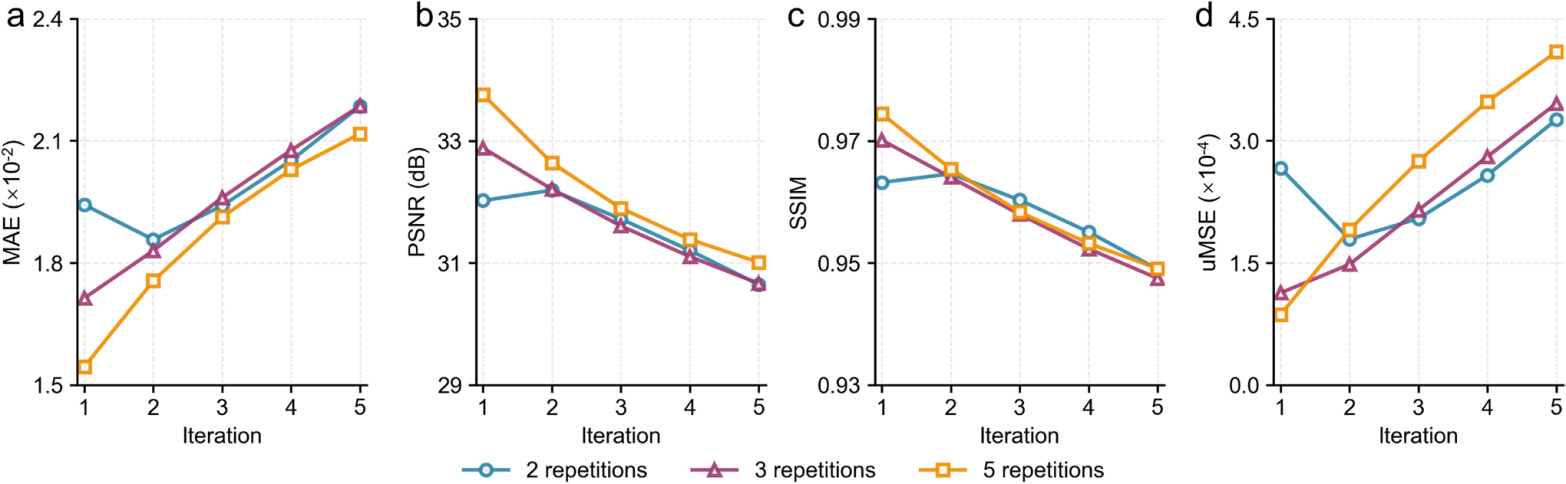
Image quality across iterations and repetitions. The performance of Noise2Average for denoising two (blue circles), three (magenta triangles), and five (orange squares) repetitions of simulated images with additive Gaussian noise (σ = 0.3) across one to five iterations is shown. Performance is quantified by the mean absolute error (MAE, a), peak signal-to-noise ratio (PSNR, b), and structural similarity index (SSIM, c) between the denoised results and the ground truth, as well as the unsupervised mean squared error (uMSE, d) calculated between the denoised image and an additional repetition of the noisy input.

The number of iterations is the only hyperparameter in Noise2Average (except for the CNN architecture and parameters), which offers a way to balance denoising strength and image sharpness. In general, Noise2Average results become cleaner after each iteration, at the cost of losing image sharpness and textural details. The optimal number of iterations depends on the number of repetitions available. For data with two repetitions, two iterations achieve the best performance. When more than two repetitions of noisy input are available, a single iteration is sufficient to achieve optimal performance (Fig. 13). For evaluation data with two noisy repetitions as the input, Noise2Average at iteration 2 achieves the highest image similarity metrics and most accurate DTI microstructural estimates compared with the ground truth (Figs. 5–10, Tables 1–6, Supplementary Figs. S1-S2, Supplementary Table S2), except for the scalar DTI metrics for the HCP-A diffusion data with a pair of noisy data with opposite phase encoding directions as the input (Figs. 7, 8, Tables 3, 4). For MGH T1w ME-MPRAGE data and OVGU 7T T1w data with more than two noisy repetitions as the input (i.e., six and eight, respectively), a single iteration is sufficient to achieve strong denoising effects and generate images with high SNR (Fig. 11). Intuitively, the result at iteration *i* (*i* = 1, 2, 3,…) is equivalent to the average of *n^i^*^+1^ raw noisy images in the ideal case (i.e., *n*^2^ at iteration 1), where *n* is the number of input noisy repetitions. Therefore, Noise2Average benefits from a larger *n*. When *n* is sufficiently large and equal to the number of repetitions acquired for generating the ground-truth high-SNR data, Noise2Average with a single iteration essentially becomes supervised learning-based denoising. Based on these results, we recommend using two iterations when working with noisy image pairs, and a single iteration when processing more than two noisy images. In cases where one needs to determine the optimal number of iterations but lacks ground-truth data, unsupervised image quality metrics, such as the unsupervised mean squared error (uMSE) (Morales et al., 2023), can be used as the stopping criterion. The calculation of uMSE requires only an additional noisy repetition and shows strong correlations with other supervised metrics computed using ground-truth data (Fig. 13).

Noise2Average has broad applications in MRI, a workhorse non-invasive imaging modality with multiple intrinsic repetitions of input data readily available for Noise2Average denoising in many scenarios. Our study demonstrates the efficacy of Noise2Average on four types of commonly acquired MRI data with two or more repetitions of noisy data as the input, including two or more consecutively acquired T1w image volumes (Figs. 5, 6, 11, Tables 1, 2), T1w image volumes with different echo times acquired using the ME-MPRAGE sequence (Fig. 6, Table 2), two diffusion image volumes acquired with opposite phase encoding directions (Figs. 7, 8, 12, Tables 3, 4), and two sets of diffusion image volumes synthesized via the diffusion tensor model from a DTI scan (Figs. 9, 10, Tables 5, 6). All these data do not require extra image co-registration or artifact correction steps and can be easily used for Noise2Average denoising. For example, the image volumes from ME-MPRAGE are inherently aligned. The diffusion image volumes acquired with opposite phase encoding directions are corrected for image intensity variation and aligned in the existing diffusion data pre-processing pipeline (e.g., using FSL’s “topup” and “eddy” functions). Many other strategies can be adopted to generate repetitions of noisy data for Noise2Average. For example, the multichannel data from phased-array coils (e.g., 32 or 64 channels) can be split and combined to form several repetitions of noisy data. Noise2Average can also be used for other imaging modalities as long as multiple repetitions of noisy input data are available.

There are several limitations. First, although validation data from five scanner types across three imaging centers demonstrate the generalizability of Noise2Average (Supplementary Table S1), a systematic cross-site and cross-scanner evaluation of existing denoising methods remains an important direction for future work to enhance clinical impact. Second, the computational cost increases with higher image resolution and a greater number of channels. Future work will explore more advanced and lightweight neural network architectures, as well as optimized fine-tuning strategies, to improve computational efficiency.

## 6 Conclusion

We propose a new iterative residual learning strategy entitled Noise2Average for denoising image data with multiple repetitions, which does not require high-SNR reference training data and can achieve self-supervision in combination with transfer learning. Noise2Average learns to map each noisy repetition to the average of all repetitions or the denoising result from the previous iteration at later iterations and recovers higher SNR by averaging CNN-denoised images at each iteration. For four types of commonly acquired T1w and diffusion MRI data, Noise2Average is quantitatively demonstrated to improve upon the classic Noise2Noise method and outperform conventional benchmark methods BM4D and AONLM, with sharper resultant images and more accurate quantitative DTI metrics that are more similar to the ground truth. Noise2Average is only slightly inferior to supervised learning-based denoising. Our data and experiments suggest that two iterations are optimal for two repetitions of input noisy data and that more repetitions of input data are recommended for which a single iteration is sufficient to achieve strong denoising effects. Without the need for high-SNR reference data, external training data, and long training time, we expect that Noise2Average can be deployed more easily in practice to benefit a broader range of clinical and neuroscientific applications that rely on highly accelerated, high-resolution, and high-SNR MRI data.

## Supplementary Material

Supplementary Material

## Data Availability

T1w and diffusion MRI data from the Human Connectome Project, WU-Minn-Ox Consortium (sections 3.1.4 and 3.2.1) and MGH-USC Consortium (section 3.2.2), are publicly available (https://www.humanconnectome.org). Diffusion MRI data from the Lifespan Human Connectome Project in Aging (section 3.1.3) are publicly available (https://www.humanconnectome.org/study/hcp-lifespan-aging). OVGU 7T T1w data (section 3.1.5) are publicly available (http://open-science.ub.ovgu.de/xmlui/handle/684882692/61). MGH gSlider-SMS diffusion MRI data (section 3.1.6) are publicly available (https://datadryad.org/stash/dataset/doi:10.5061/dryad.nzs7h44q2 and https://datadryad.org/stash/dataset/doi:10.5061/dryad.rjdfn2z8g). MGH T1w Wave-MPRAGE data (section 3.1.1) and ME-MPRAGE data (section 3.1.2) are available from the corresponding author upon reasonable request. Comprehensive diffusion MRI dataset (CDMD) is publicly available (https://doi.org/10.6084/m9.figshare.16910290) (section 3.1.7). The source codes of BM4D are publicly available (https://webpages.tuni.fi/foi/GCF-BM3D/). Details of the MATLAB-based AONLM software package are available at https://www.nitrc.org/projects/mri-denoising. The package is accessible by contacting either the corresponding author or the developers (Manjón et al., 2010). The source codes of Noise2Noise and Noise2Average implemented using Keras application programming interface is publicly available (https://github.com/birthlab/Noise2Average).
