# Supplementary material for: Noise2Average: An iterative residual learning strategy for image denoising without clean data
Source: Imaging Neurosci (Camb). 2026 Mar 24;4:IMAG.a.1163. doi: 10.1162/IMAG.a.1163 (PMC13015446; doi:10.1162/IMAG.a.1163)
Supplement: Supplementary Material [file IMAG.a.1163_supp.pdf]

Noise2Average: an iterative residual learning strategy for image denoising without clean data

Zihan Li[1], Ziyu Li[2], Berkin Bilgic[3,4,5], Kui Ying[6], David H. Salat[3,4],
Jonathan R. Polimeni[3,4,5], Hongen Liao[1], Susie Y. Huang[3,4,5], Qiyuan Tian[1*]

[1]School of Biomedical Engineering, Tsinghua University, Beijing, P.R. China;
[2]Oxford Centre for Integrative Neuroimaging, FMRIB, Nuffield Department of Clinical Neurosciences, University of Oxford, Oxford, UK;
[3]Athinoula A. Martinos Center for Biomedical Imaging, Department of Radiology, Massachusetts General Hospital, Charlestown, MA, USA;
[4]Department of Radiology, Harvard Medical School, Boston, MA, USA;
[5]Harvard-MIT Program in Health Sciences and Technology, Massachusetts Institute of Technology, Cambridge, MA, USA;
[6]Department of Engineering Physics, Tsinghua University, Beijing, P.R. China.

[*]Correspondence to: Qiyuan Tian, Ph.D., Center for Biomedical Imaging Research at Tsinghua University, 30 Shuangqing Road, Haidian District, Beijing, China, 100084. E-mail: qiyuantian@tsinghua.edu.cn.

Supplementary Materials

Derivations for Noise2Average

Let $[x_1, x_2, \ldots, x_N]$ be *N* noisy images, where $x_i = s + n_i$, with s as the noise-free image, $n_i \sim \mathcal{N}(0, \sigma^2 \mathrm{I})$ as independent and identically distributed additive Gaussian white noise. Noise2Average proceeds with an initial estimate computed as the simple average:

$$\widehat{s_0} = \frac{1}{N}\sum_{i=1}^{N} x_i = s + \frac{1}{N}\sum_{i=1}^{N} n_i \tag{1}$$

$$E[\widehat{s_0}] = s, Var(\widehat{s_0}) = \frac{\sigma^2}{N}. \tag{2}$$

At each iteration k, the training objective is to minimize:

$$arg\,\min_{\theta_k} \frac{1}{N}\sum_{i=1}^{N} E\left[\left|\left|f_{\theta_k}(x_i) - \hat{s}_{k-1}\right|\right|_2^2\right]. \tag{3}$$

According to Noise2Noise principle, when using MSE loss, the optimal solution is the conditional expectation:

$$f_{\theta_k}^{*}(x_i) = E[\hat{s}_{k-1} | x_i]. \tag{4}$$

Taking the first iteration as an example, the optimal mapping becomes:

$$f_{\theta_1}^{*}(x_i) = E[\hat{s}_0 | x_i] = s + \frac{1}{N} n_i. \tag{5}$$

Therefore, the result of the first iteration, which becomes the target for the next iteration is:

$$\widehat{s_1} = \frac{1}{N}\sum_{i=1}^{N} f_{\theta_1}^{*}(x_i) = s + \frac{1}{N^2}\sum_{i=1}^{N} n_i \tag{6}$$

$$E[\widehat{s_1}] = s, Var(\widehat{s_1}) = \frac{\sigma^2}{N^3}. \tag{7}$$

Similarly, the result for the second iteration ($k = 2$) becomes:

$$f^*_{\theta_2}(x_i) = E[\widehat{s_1}|x_i] = s + \frac{1}{N^2} n_i \tag{8}$$

$$\widehat{s_2} = \frac{1}{N}\sum_{i=1}^{N} f^*_{\theta_2}(x_i) = s + \frac{1}{N^3}\sum_{i=1}^{N} n_i \tag{9}$$

$$E[\widehat{s_2}] = s, Var(\widehat{s_2}) = \frac{\sigma^2 + \delta^2}{N^5}. \tag{10}$$

After k iterations, the optimal Noise2Average output $\widehat{s_k}$ becomes:

$$E[\widehat{s_k}] = s, Var(\widehat{s_k}) = \frac{\sigma^2}{N^{2k+1}}. \tag{11}$$

According to the Noise2Noise principle, $E[\widehat{s_k}]$ serves as the equivalent training target for Equation 3. Therefore, $E[\widehat{s_k}] = s$, converging to ground truth with exponentially reduced variance $Var(\widehat{s_k}) = \frac{\sigma^2}{N^{2k+1}}$.

Dataset summary

Table S1. Data summary. Data acquisition scanners, sites, and availability for datasets used in this study are listed.

Data	Scanner	Site	Availability
Wave-MPRAGE data	3T Skyra and 3T Prisma	Massachusetts General Hospital	Private
ME-MPRAGE data	3T Trio Tim	Massachusetts General Hospital	Private
HCP-A data	3T Prisma	Massachusetts General Hospital	Public
WU-Minn-Ox HCP data	3T Skyra	Washington University	Public
OVGU data	7T MAGNETOM	Otto-von-Guericke University	Public
gSlider-SMS data	3T Connectom	Massachusetts General Hospital	Public

Simulation experiment

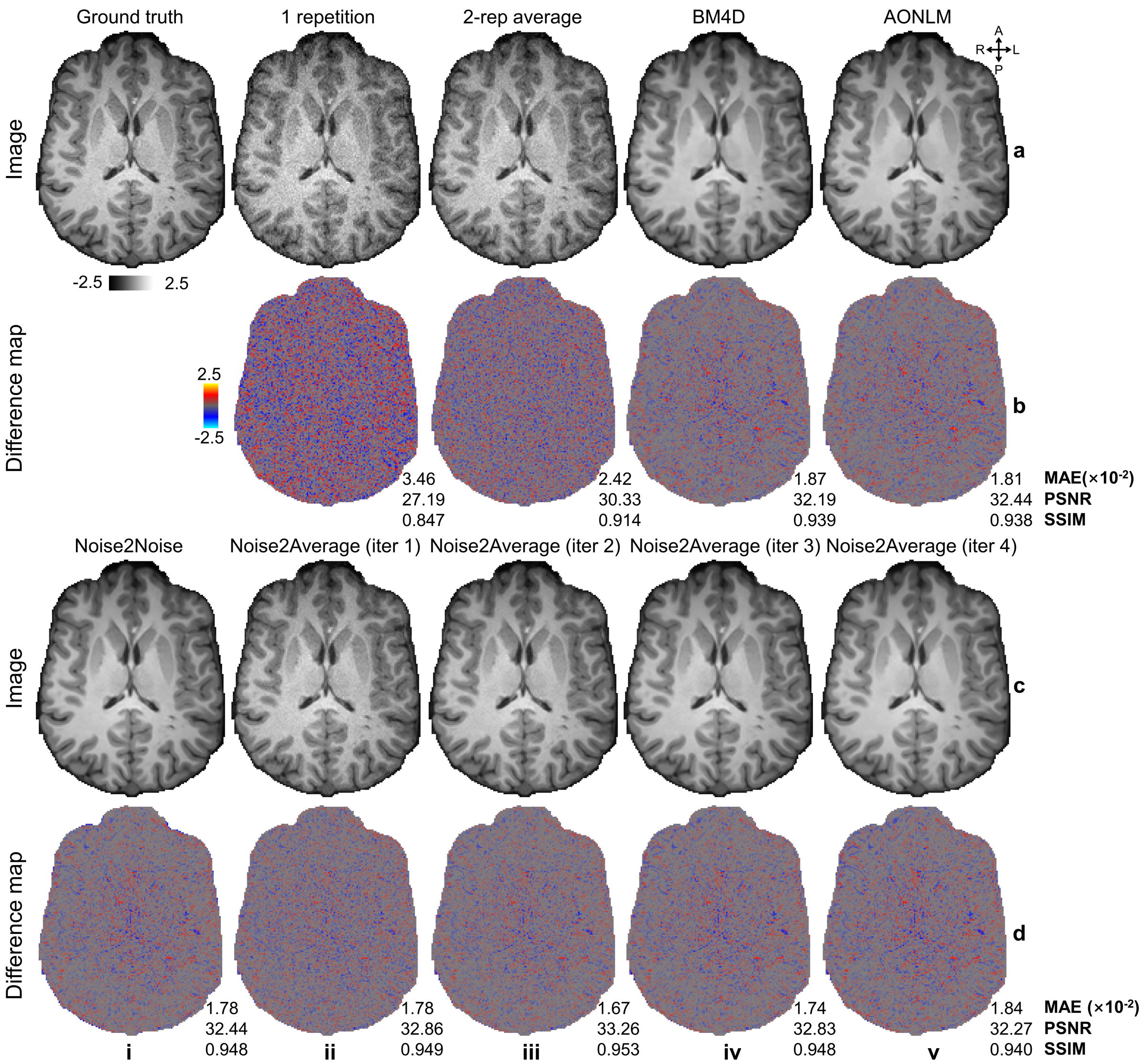

Figure S1. Image results of simulated data with mild noise. Exemplary axial image slices from the ground-truth image volume (a, i), single noisy image volume with simulated added Gaussian noise (a, ii, noise level σ=0.3), two-repetition averaged image volume (a, iii), BM4D-denoised two-repetition averaged volume (a, iv), AONLM-denoised two-repetition averaged volume (a, v), Noise2Noise-denoised data (c, i), and Noise2Average-denoised data from iteration 1 to 4 (c, ii-v) of a representative subject from CDMD dataset are shown, along with the difference maps compared to the ground truth (b, d). Mean absolute error (MAE), peak SNR (PSNR), and structural similarity index (SSIM) are listed to quantify the similarity between different images and the ground truth.

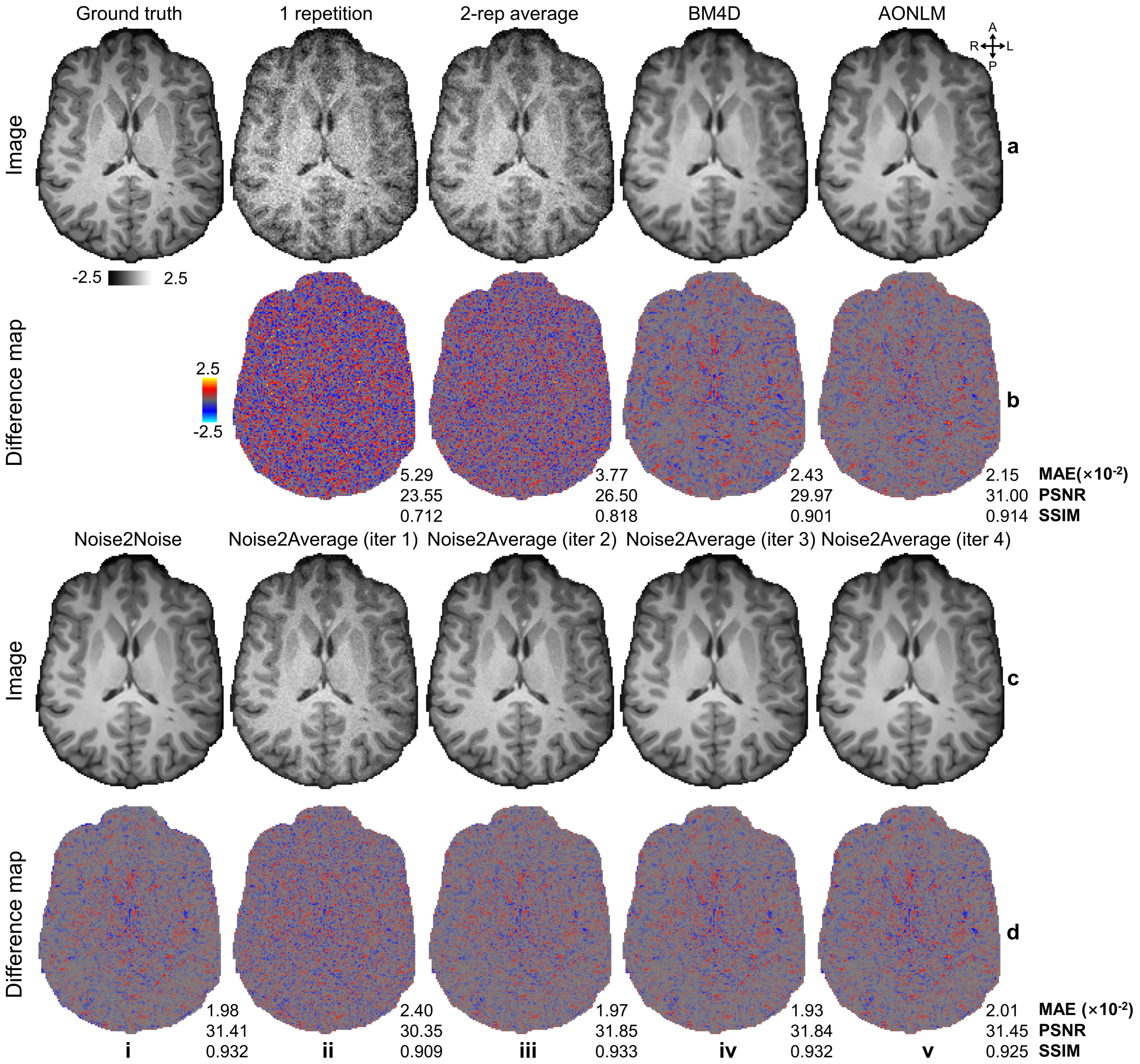

Figure S2. Image results of simulated data with heavy noise. Exemplary axial image slices from the ground-truth image volume (a, i), single noisy image volume with simulated added Gaussian noise (a, ii, noise level σ=0.5), two-repetition averaged image volume (a, iii), BM4D-denoised two-repetition averaged volume (a, iv), AONLM-denoised two-repetition averaged volume (a, v), Noise2Noise-denoised data (c, i), and Noise2Average-denoised data from iteration 1 to 4 (c, ii-v) of a representative subject from CDMD dataset are shown, along with the difference map compared to the ground truth (b, d). Mean absolute error (MAE), peak SNR (PSNR) and structural similarity index (SSIM) are listed to quantify the similarity between different images and the ground truth.

Table S2. Image metrics of simulated data. The group means (± group standard deviations) of the mean absolute error (MAE), peak signal-to-noise ratio (PSNR), and structural similarity index (SSIM) between the single noisy image volume with simulated added Gaussian noise (a: σ=0.3, k: σ=0.5), two-repetition averaged image volume (b, l), BM4D-denoised two-repetition averaged volume (c, m), AONLM-denoised two-repetition averaged volume (d, n), Noise2Noise-denoised data (e, o), and Noise2Average (N2A)-denoised data from iteration 1 to 5 (f-i, p-t) and the ground truth image volume of 10 evaluation subjects from CDMD dataset are listed. Red and green texts indicate the best and the second-best performance from different methods. Comparisons between N2A (iter2) and other methods were performed using paired t-test (***: p<0.001, **: p<0.01, *: p<0.05)

**Gaussian noise**	a	b	c	d	e
**Mild: σ=0.3**	1-rep	2-rep avg	BM4D	AONLM	Noise2Noise
MAE (×10^{-2})	3.434±0.1369***	2.525±0.1108***	1.910±0.1190***	1.863±0.1131***	1.866±0.1398***
PSNR (dB)	27.27±0.3804***	29.93±0.4203***	31.92±0.5500***	32.13±0.5550***	32.03±0.7007***
SSIM	0.850±0.0166***	0.909±0.0093***	0.939±0.0054***	0.939±0.0060***	0.945±0.0062***
	f	g	h	i	j
	N2A (iter 1)	N2A (iter 2)	N2A (iter 3)	N2A (iter 4)	N2A (iter 5)
MAE (×10^{-2})	1.896±0.1231***	1.779±0.1351	1.841±0.1441***	1.940±0.1521***	2.038±0.1661***
PSNR (dB)	32.24±0.6462***	32.61±0.7398	32.25±0.7429***	31.77±0.7285***	31.34±0.7453***
SSIM	0.945±0.0062***	0.949±0.0059	0.944±0.0065***	0.937±0.0075***	0.930±0.0087***
**Gaussian noise**	k	l	m	n	o
**Heavy: σ=0.5**	1-rep	2-rep avg	BM4D	AONLM	Noise2Noise
MAE (×10^{-2})	5.283±0.0166***	3.789±0.0314***	2.362±0.1028***	2.110±0.0874***	1.993±0.1106
PSNR (dB)	23.55±0.0266***	26.45±0.0743***	30.22±0.3470***	31.13±0.3282***	31.42±0.4630***
SSIM	0.718±0.0205***	0.819±0.0139***	0.909±0.0064***	0.921±0.0061***	0.933±0.0059
	p	q	r	s	t
	N2A (iter 1)	N2A (iter 2)	N2A (iter 3)	N2A (iter 4)	N2A (iter 5)
MAE (×10^{-2})	2.419±0.0559***	1.988±0.0814	1.958±0.1003***	2.035±0.1114***	2.127±0.1203***
PSNR (dB)	30.27±0.2097***	31.74±0.3534	31.72±0.4222	31.33±0.4433***	30.93±0.4544***
SSIM	0.908±0.0069***	0.933±0.0051	0.933±0.0054	0.927±0.0060***	0.919±0.0066***

Pre-training strategy comparison

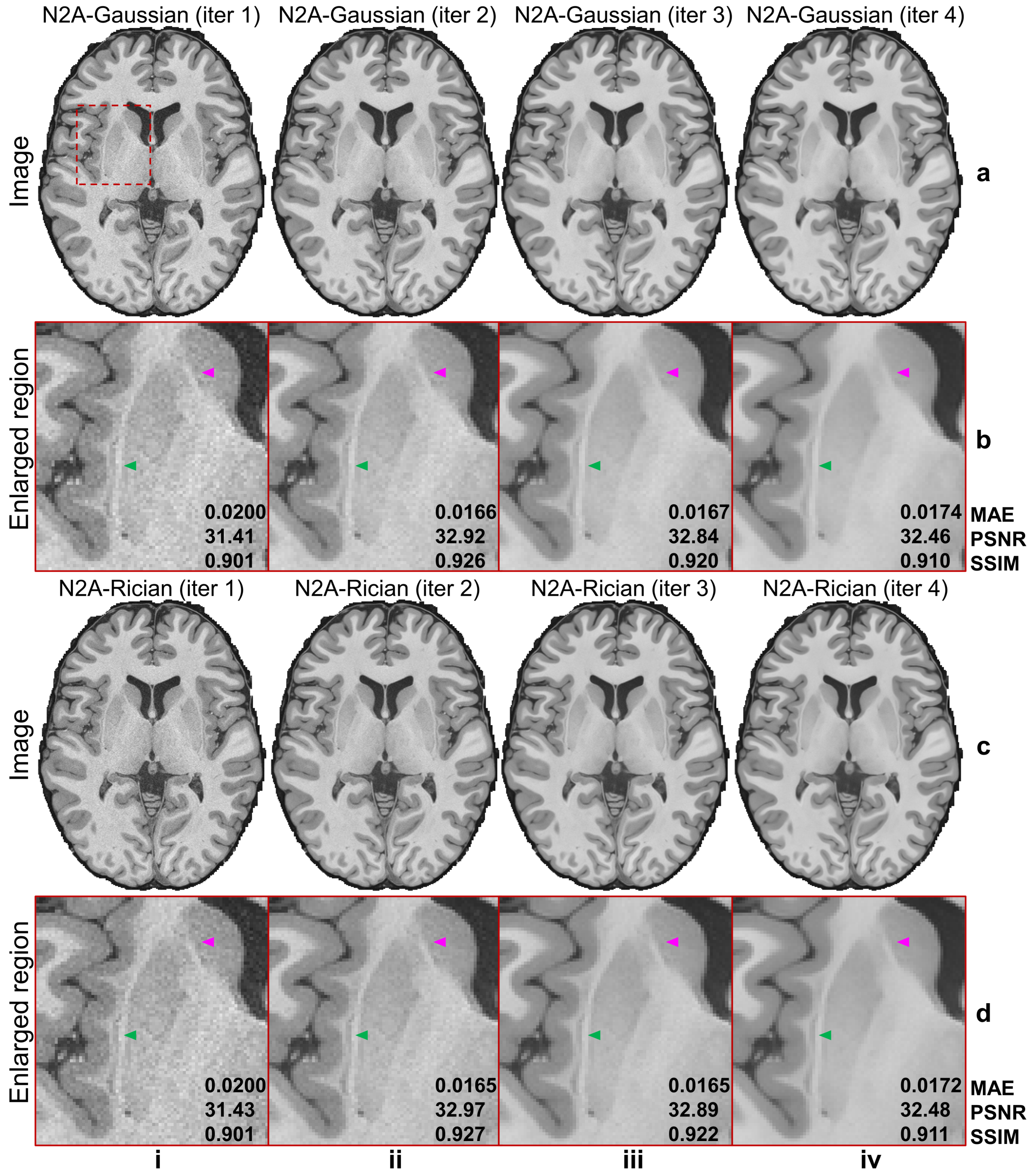

Figure S3. Comparison of pre-training strategy. Exemplary axial image slices of T1w MRI data denoised using Noise2Average (N2A) are shown. The models were pre-trained on simulated T1w MRI data with either Gaussian noise (a-b, copied from Fig. 6) or Rician noise (c-d), respectively. For a fair comparison, both simulated datasets contained 20 subjects and the PSNR of simulated noisy inputs was 23 dB. Mean absolute error (MAE), peak signal-to-noise ratio (PSNR), and structural similarity index (SSIM) values quantify the similarity between each denoised image and the ground truth.
